# Hypoglycemia induces brain metabolic reprogramming and neurodegeneration via serum response factor and myocardin-related transcription factor-A

**DOI:** 10.1038/s41392-025-02527-x

**Published:** 2025-12-17

**Authors:** Minjeong Jang, Hyung Jin Choi, Hae-June Lee, Hong Nam Kim

**Affiliations:** 1 Divisions of Radiation Biomedical Research, Korea Institute of Radiological and Medical Sciences (KIRAMS), Seoul, Republic of Korea; 2 Department of Brain and Cognitive Sciences, Department of Anatomy and Cell Biology, Neuroscience Research Institute, Wide River Institute of Immunology, Seoul National University, Seoul, Republic of Korea; 3 College of Veterinary Medicine, Jeju National University, Jeju, Republic of Korea; 4 Brain Science Institute, Korea Institute of Science and Technology (KIST), Seoul, Republic of Korea; 5 Division of Bio-Medical Science & Technology, KIST School, Korea University of Science and Technology (UST), Seoul, Republic of Korea; 6 School of Mechanical Engineering, Yonsei University, Seoul, Republic of Korea; 7 Yonsei-KIST Convergence Research Institute, Yonsei University, Seoul, Republic of Korea

**Keywords:** Cellular neuroscience, Diseases of the nervous system

## Abstract

Hypoglycemia is a frequent and potentially severe complication that can result in significant brain injury in individuals with diabetes treated with insulin or other hypoglycemic agents and in those undergoing prolonged fasting. Despite its clinical importance, the molecular mechanisms through which hypoglycemia induces neurodegeneration remain poorly defined. We therefore investigated the molecular and cellular basis of hypoglycemia-induced brain damage using human neuron and glial cell cultures in vitro and hypoglycemic mouse models in vivo. We found that starvation-induced hypoglycemia triggers hallmark neurodegenerative features, such as astrocyte activation and microglial reactivity, that closely resemble those found in the brains of hypoglycemic mouse models. Neurons notably activate an adaptive survival response mediated by serum response factor (SRF) and myocardin-related transcription factor-A (MRTF-A), which drives a metabolic reprogramming process. This shift enables neurons to use extracellular matrix components as alternative energy sources under glucose deprivation. However, this compensatory mechanism results in the excessive accumulation of urea cycle byproducts, which subsequently exacerbates neuronal damage and promotes glial activation. Glucose refeeding remarkably reversed these neurodegenerative features by deactivating SRF/MRTF-A signaling in both in vitro and in vivo. Collectively, our results revealed a neuron-intrinsic mechanism linking glucose deprivation to reversible neurodegeneration *via* SRF/MRTF-A, offering potential targets for preventing hypoglycemia-associated brain damage.

## Introduction

Hypoglycemia, defined as a reduction in blood glucose levels below 70 mg/dL,^1^ is a common metabolic condition resulting from impaired glucose homeostasis.^2^ It is most frequently observed in individuals with diabetes under therapy with insulin or hypoglycemic agents, but it can also affect non-diabetic individuals during prolonged fasting, malnutrition, or specific medical conditions.^1–4^ Dietary interventions such as caloric restriction (CR) and intermittent fasting have recently attracted considerable attention for their potential to extend lifespans, delay age-related pathologies, and confer neuroprotective effects.^5–7^ However, severe or prolonged CR is associated with substantial risks such as hypoglycemia, metabolic imbalance, and increased mortality rates in severe energy-deficient states such as anorexia nervosa.^8,9^ Despite the widespread adoption of fasting and energy-limiting interventions, the neurobiological consequences of recurrent or sustained hypoglycemia remain poorly defined.^10^ Recurrent or sustained hypoglycemic episodes, whether diet- or therapy-induced, might therefore impose cumulative stress on neuronal circuits, underscoring a critical need to elucidate the cellular and molecular mechanisms underlying neuron-specific adaptation and injury in response to insufficient energy.

Glucose is the primary energy substrate for the brain, which relies on a continuous supply to sustain critical neuronal functions, including synaptic transmission, plasticity, and the metabolic support provided by astrocytes and other glial populations.^11^ Given the high energy demand in the brain,^12^ electrically active and post-mitotic neurons are acutely sensitive and susceptible to even transient reductions in glucose availability, which can impair synaptic transmission and plasticity. Disrupted glucose metabolism can therefore lead to a cascade of neurological consequences ranging from acute cognitive impairment to progressive neurodegeneration. Increasing epidemiological and experimental evidence has associated impaired glucose homeostasis with elevated susceptibility to neurodegenerative disorders such as Alzheimer’s disease (AD) and Parkinson’s disease (PD).^12–15^ Despite these associations, the underlying cellular and molecular mechanisms through which glucose deprivation promotes neurodegenerative processes remain poorly defined. While hyperglycemia and its complications in the brain have been extensively studied,^16^ the neurological consequences of hypoglycemia remain comparatively underexplored, particularly regarding its effects on neuronal metabolism, transcriptional regulation, and synaptic maintenance. Defining how energy deficits perturb neuronal networks is therefore essential for understanding how metabolic stress accelerates neurodegenerative processes. Such insights will be critical for developing targeted interventions to preserve brain integrity in individuals exposed to recurrent or sustained hypoglycemia, whether through insulin therapy, strict CR, or intermittent fasting.

Although brain dysfunction has been associated with hypoglycemia,^17,18^ studies have predominantly relied on systemic animal models or clinical findings, which limit insight into the contributions of individual cell types and the local microenvironment. In particular, the cell-type specific molecular mechanisms of individual brain cell populations, such as neurons, astrocytes, and microglia, remain poorly defined in the context of hypoglycemic stress. Moreover, the impact of nutrient scarcity on brain metabolism at the microenvironmental level, including extracellular matrix (ECM) remodeling, local metabolic interactions, and signaling networks that coordinate adaptive or pathological responses, has been rarely assessed. The transcriptional regulators that mediate neuronal survival or trigger neurodegeneration under glucose-deprived conditions remain poorly characterized, representing a critical knowledge gap in understanding hypoglycemia-associated neurodegeneration. To address these gaps, physiologically relevant platforms in vitro are required to decouple systemic effects from neuron-intrinsic responses, enabling detailed investigations of candidate regulatory pathways under controlled conditions.^19^ Accumulating evidence indicates that brain-derived cells cultured in physiologically relevant three-dimensional (3D) environments, such as hydrogel-embedded systems, display phenotypes such as divergent marker expression, migratory behavior, and transcriptomic profiles that differ from two-dimensional (2D) cultures.^20,21^ Further, changes in the composition of the surrounding extracellular matrix can drive epigenetic remodeling, such as histone acetylation and DNA methylation.^20,22^ These models can also provide insights into neuron-glial crosstalk, metabolic reprogramming, and ECM dynamics during energy stress, which are challenging to investigate in vivo owing to systemic confounding factors.

Here, we employed integrative approaches using 3D neuronal cultures in vitro and mouse models of hypoglycemia in vivo to dissect the cellular and molecular consequences of glucose deprivation-induced neurodegeneration. These complementary models enabled us to explore whether hypoglycemia elicits the canonical hallmarks of neurodegeneration, namely amyloid accumulation, tau hyperphosphorylation, reactive astrocytosis, microglial activation, and neuronal metabolic reprogramming. Our findings revealed that neurons adapt to glucose-depleted conditions by using alternative energy sources, which sustain energy production and survival. In particular, we focused on uncovering the transcriptional networks and metabolic adaptations that govern neuronal responses to glucose deprivation, emphasizing the roles of the transcription factor serum response factor (SRF) and its coactivator, myocardin-related transcription factor-A (MRTF-A), as key regulators. Activation of the SRF/MRTF-A axis orchestrates broad transcriptional programs that coordinate ECM utilization, amino acid metabolism, and stress response pathways, which effectively balance neuronal survival with susceptibility to neurodegeneration. Furthermore, restoring glucose reverses neurodegenerative changes caused by hypoglycemia, with a gradual decrease in SRF/MRTF-A transcriptional activity, highlighting a strong potential for neuronal recovery. Collectively, these findings elucidated the molecular mechanisms linking energy deprivation to neurodegenerative processes, providing a powerful framework for potential therapeutic strategies to preserve neuronal function during metabolic stress.

## Results

### Long-term starvation induces neurodegenerative pathology in mouse brains

**Fig. 1:**
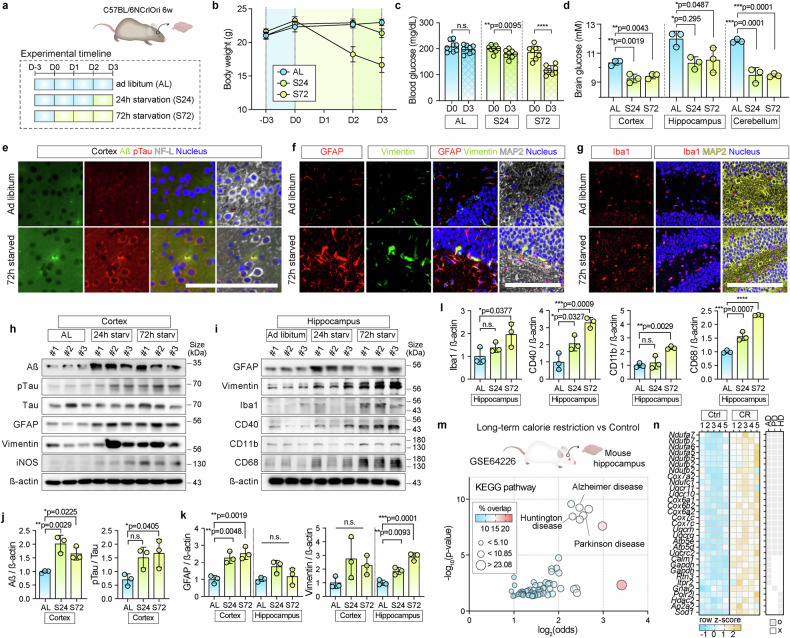
Long-term starvation induces neurodegenerative pathology in mice. **a** Experimental timeline for hypoglycemic mouse models in vivo. Male C57BL/6 mice were subjected to starvation for 24 or 72 h with access to water only. **b** Body weight measurements under (i) ad libitum (AL), (ii) 24-h starvation (S24), and (iii) 72-hour starvation (S72) conditions. Body weight was measured under all experimental conditions (*n* = 8; ****p < 0.0001). **c** Blood glucose levels under time-variable starvation. Blood glucose level was measured before and after the starvation. Statistical significance was determined using an unpaired two-tailed t-test (*n* = 8; ****p < 0.0001, n.s. = not significant). **d** Intracellular glucose levels measured in the cerebral cortex, hippocampus, and cerebellum following starvation (*n* = 3). **e** Immunofluorescence stained Aβ (green) and pTau (red) in the cerebral cortex under AL and S72. Nuclei (blue) and NF-L (white) were co-stained. Scale bar = 100 µm. **f** Immunofluorescence staining of GFAP (red) and vimentin (green) in hippocampal dentate gyrus (DG) under AL and S72. MAP2 (white) and nuclei (blue) were co-stained. Scale bar = 100 µm. **g** Immunofluorescence staining of Iba1 (red) in the hippocampal DG under AL and S72 conditions. Nuclei (blue) and MAP2 (yellow) were co-stained. Scale bar = 100 µm. Western blots of **h** Aß, pTau, total Tau, GFAP, vimentin, and iNOS in the cerebral cortex and **i** GFAP, vimentin, Iba1, CD40, CD11b, and CD68 in the hippocampus under AL, S24, and S72. β-Actin was used as a loading control. **j**–**l** Quantification of relative protein expression levels compared to AL conditions in the cerebral cortex and hippocampus regions in the mouse brains. **j** Aß and pTau/Tau ratio in the cerebral. **k** GFAP expression in both the cerebral cortex and the hippocampus. **l** Expression levels of Iba1, CD40, CD11b, and CD68 in the hippocampus under AL, S24, and S72 conditions. Data are presented as a scatter dot plot (mean ± SD, *n* = 3; ****p < 0.0001, n.s.; not significant). Statistical significance in (**d**), (**j**), (**k**), and (**l**) was calculated using an ordinary one-way analysis of variance, followed by Tukey’s multiple comparison test. **m** Gene Ontology analysis of significantly upregulated genes (≥1.5-fold) in mouse hippocampus under long-term calorie-restriction using GSE64226 (*n* = 5 per group). KEGG pathway terms are shown as odds ratios with *p* < 0.05. **n** Heatmap of differentially expressed genes associated with AD, PD, and HD-related genes from Kyoto Encyclopedia of Genes and Genomes (KEGG) pathway was generated based on row *z-*scores. The genes included in each gene set—AD, HD, and PD—are presented in gray

We established a mouse model in vivo to validate the molecular characteristics of neurodegeneration induced by hypoglycemia. We analyzed blood glucose levels and brain tissues in mice after a 24- or 72-h fast with access to water (Fig. 1a). Starvation for 24 and 72 h resulted in a significant reduction in body weight (Fig. 1b), accompanied by a decline in blood glucose levels from 180.8 (control) to 180.38 and 127.8 mg/dL, respectively (Fig. 1c). Glucose concentrations significantly decreased in the cerebral cortex, hippocampus, and cerebellum (Fig. 1d).

Notably, we observed pronounced neurodegenerative changes under hypoglycemic conditions, which became more severe with extended starvation (Fig. 1e–l, Supplementary Fig. 1). Specifically, expression of the neurodegenerative markers amyloid-beta (Aß) and phosphorylated Tau (pTau),^23^ and astrocyte activation markers such as glial fibrillary acidic protein (GFAP) and vimentin were elevated,^24,25^ in the cortex, hippocampus, and cerebellum (Fig. 1e, f, h–k, Supplementary Fig. 1). The expression of Aß42 was significantly increased under starvation, rather than Aß40, in the cerebral cortex (Supplementary Fig. 1d); Aß42 is more likely to form aggregates than Aß40, which is an AD marker.^26^ Microglial activation markers, including Iba1, CD40, CD11b, and CD68,^27,28^ were significantly upregulated in brains under fasting (Fig. 1g, i, l, Supplementary Fig. 1), indicating a robust glial response to prolonged glucose deprivation. Moreover, the neuroinflammatory marker inducible nitric oxide synthase (iNOS),^29^ which is mainly expressed in astrocytes and microglia, was significantly upregulated in all examined brain regions (Fig. 1h, Supplementary Fig. 1).

We compared hippocampal gene expression in mice under long-term CR with controls using information downloaded from the Gene Expression Omnibus (GEO) database (Fig. 1m, n). We identified 566 genes that were upregulated >1.5-fold in the CR, compared to the control group. Gene set enrichment analysis (GSEA) revealed that the upregulated genes were significantly associated with pathways associated with neurodegeneration in AD, Huntington’s disease (HD), and PD (Fig. 1m). Furthermore, numerous individual neurodegenerative genes were upregulated in the hippocampi of CR mice (Fig. 1n), supporting an association between sustained nutrient deprivation and neurodegenerative molecular signatures.

### Development of a hypoglycemic model in vitro

**Fig. 2:**
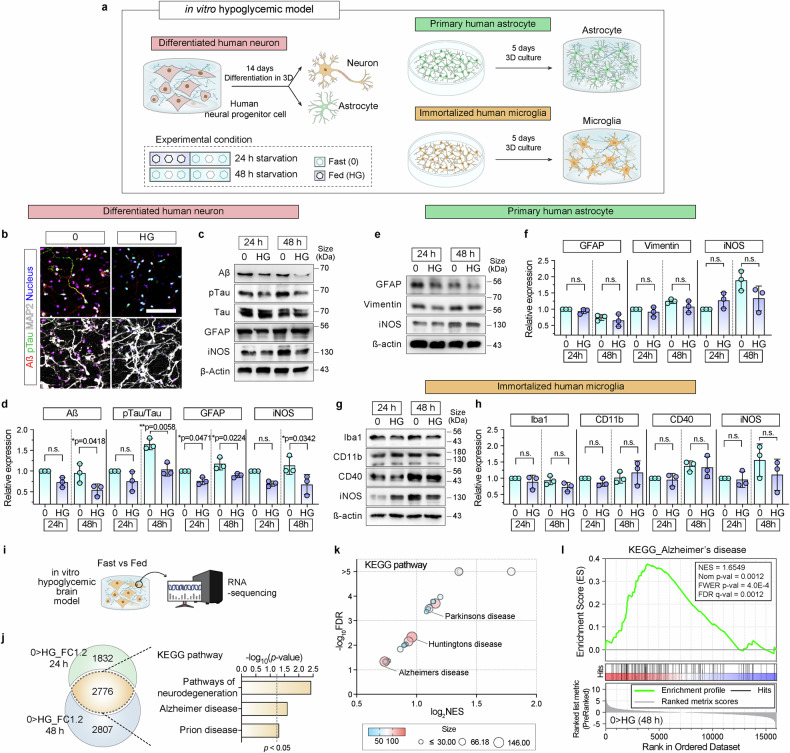
Hypoglycemia induces neurodegeneration in 3D hypoglycemic models in vitro. **a** Schematic illustration of 3D hypoglycemic model in vitro. Human neural progenitor cells (HN) were differentiated into astrocytes and neurons for 14 days in a 3D hydrogel scaffold consisting of Matrigel and collagen type I. Primary human astrocytes (HA) and immortalized human microglia (HM) were cultured for 5 days in the same matrix. After differentiation and culture, hypoglycemic conditions were induced by treating cells with a glucose-free medium for 24 or 48 h. **b** Immunofluorescence staining of MAP2 (white), Aβ (red), and pTau (green) in differentiated HN under glucose-starved (0) and fed (HG) conditions after 48 h. Nuclei (blue) were stained with DAPI. Scale bar = 100 µm. **c**, **e**, **g** Western blot analysis of protein markers in HN (**c**), HA (**e**), and HM (**g**) cultured in 3D under 0 and HG conditions for 24 and 48 h. **c** Neurodegenerative markers: Aß, pTau, and Tau; reactive astrocyte marker: GFAP; and neuroinflammation marker: iNOS. **e** Reactive astrocyte markers: GFAP and vimentin, and iNOS. **g** Microglial activation markers: Iba1, CD11b, CD40, and iNOS. ß-actin was used as a loading control. **d**, **f**, **h** Protein levels in (**c**), (**e**), and (**g**) were quantified and normalized to glucose depletion for 24 h. Graphs represented the relative expression values of neurodegenerative and neuroinflammatory markers (**d** Aß, ratio of pTau/Tau, and iNOS), reactive astrocyte markers (**f** GFAP, vimentin, and iNOS), and microglial activation markers (**h** Iba1, CD11b, CD40, and iNOS) proteins under 0 and HG conditions for 24 and 48 h. The scatter dot plot in (**d**), (**f**) and (**h**) represents the mean ± SD with bars and error bars. Statistical significance was calculated using an unpaired two-tailed t-test (*n* = 3; n.s.; not significant). **i** RNA sequencing of differentiated HN under 0 and HG conditions for 24 and 48 h. **j** Venn diagram of 2775 genes upregulated ≥1.2-fold in starved cells at both 24 and 48 h compared with those in HG cells. Gene Ontology analysis of overlapping genes from KEGG pathways. Dashed vertical line indicates *p* < 0.05 threshold. **k** Gene set enrichment analysis shows significantly enriched KEGG pathways under hypoglycemia. Bubble plot represents significantly enriched gene sets with FDR < 0.05 and NES as log_2_ value. **l** Enrichment plot of the AD gene set from KEGG shows significant enrichment in the starved group relative to the fed group. Red and blue indicate high- and low-ranked genes, respectively. FDR false discovery rate, NES normalized enrichment score

We developed hypoglycemic models in vitro using 2D and 3D culture systems to investigate the molecular mechanisms underlying neurodegeneration induced by long-term starvation (Fig. 2a, Supplementary Fig. 2a, g, k). The 3D models were made of a hybrid ECM consisting of type I collagen and Matrigel, and its effects were compared with those of conventional 2D monolayer cultures on the specific responses of human neural progenitor (ReN) cells (referred to as HN), primary astrocytes (HA), and immortalized microglia (HM). We found that HN differentiated into neurons and astrocytes^30^ over 14 days in the 3D culture system (Fig. 2a, Supplementary Figs. 2a and 3), whereas HA and HM cells were incubated for 5 days under the same conditions in the 2D and 3D culture systems (Fig. 2a, Supplementary Fig. 2g, k). Hypoglycemic stress was induced by exposing the cultures to glucose-depleted medium for 24 (short-term) and 48 h (long-term) (Fig. 2a, Supplementary Fig. 2a, g, k). We performed RNA sequencing and bioinformatics analysis to evaluate the molecular biology of 2D and 3D hypoglycemic models, elucidating the neurodegenerative mechanisms induced by starvation.

### Neuronal cell viability was maintained in 3D hypoglycemic models

Differentiated human neurons cultured in the composite 3D hydrogel maintained high viability even after 48 h of glucose starvation (Supplementary Fig. 2b–d). In contrast, most HN cultured under 2D conditions without ECM support did not survive glucose deprivation for 48 h (Supplementary Fig. 2b). Primary astrocytes retained their viability in the 3D matrix after starvation, whereas 2D-cultured astrocytes exhibited substantial cell death after 48 h (Supplementary Fig. 2h). The viability of both 2D- and 3D-cultured HM was significantly diminished under prolonged glucose starvation (Supplementary Fig. 2l). These findings suggested that the ECM plays a crucial role in supporting neuronal cell survival under hypoglycemia, whereas its protective effects seem to be limited to microglia.

### Glucose starvation induces neurodegeneration in a 3D hypoglycemic model in vitro

Consistent with our findings in starved mouse brains, the hallmarks of neurodegeneration were also found in differentiated human neurons under glucose starvation (0) in the 3D model. Increased Aβ and pTau expression indicated AD-like pathology (Fig. 2b–d, Supplementary Fig. 2e), and iNOS and GFAP levels were significantly elevated after 24 and 48 h of glucose deprivation (Fig. 2c, d, Supplementary Fig. 2e).

In contrast, primary human astrocytes exhibited no substantial changes in the expression of reactive astrocyte markers (GFAP, vimentin, and iNOS) under similar glucose starvation conditions (Fig. 2e, f, Supplementary Fig. 2i). Glucose deprivation for 24 and 48 h also minimally affected microglial activation markers, such as Iba1, CD11b, CD40, and iNOS in HM (Fig. 2g, h, Supplementary Fig. 2m). Furthermore, no significant neurodegenerative changes were observed in any of the three cell types (differentiated HN, primary HA, and immortalized HM) after 24 h of glucose deprivation in the 2D hypoglycemic model (Supplementary Fig. 2f, j, n).

We performed RNA sequencing of differentiated human neurons under starvation (0) and high-glucose conditions at 24 and 48 h to gain deeper molecular insights. We identified 2776 genes that were upregulated >1.2-fold under starvation (0) (Fig. 2i). Analysis of Kyoto Encyclopedia of Genes and Genomes (KEGG) pathways revealed that these genes were significantly associated with neurodegenerative pathways, including AD and prion diseases (Fig. 2j). Additionally, GSEA findings indicated that gene sets associated with AD, PD, and HD were enriched under glucose starvation for 48 h, compared with controls that were not starved (Fig. 2k).

### Starved neurons used the ECM for their survival

The viability of differentiated HN and primary HA cultured in 3D matrix-embedded systems was sustained under glucose starvation, but not in immortalized HM. In contrast, hypoglycemic stress resulted in significant death among the three cell types cultured under 2D conditions. These findings led us to hypothesize that ECM components serve as alternative energy sources for neurons during hypoglycemia, and therefore potentially contribute to neuronal survival.

We cultured HN, HA, and HM cells with the ECM components DQ-collagen types I and IV in 2D models to test this hypothesis (Supplementary Fig. 4a). We used DQ-collagen IV and collagen-1 that emit fluorescence when enzymatically degraded to directly monitored ECM utilization (Supplementary Fig. 4b). Notably, the viability of HN cells was significantly improved under collagen I- and IV-treated conditions after 24 h of glucose starvation, with collagen IV exhibiting a strong protective effect after 48 h (Supplementary Fig. 4c). Collagen IV similarly improved the viability of HA and HM, whereas collagen I exerted a mild beneficial effect on astrocytes and a more significant impact on microglial survival under starvation (Supplementary Fig. 4d, e).

We quantified the collagen content of the culture media and lysates of 2D-cultured HN, HA, and HM after 24 h of glucose deprivation to evaluate ECM utilization (Supplementary Fig. 4f–i). In the culture media, collagen type I levels showed a slight, non-significant decrease in HN and HM but were significantly elevated in HA under starvation conditions. Conversely, collagen type IV levels decreased substantially in all cell types, indicating its active utilization (Supplementary Fig. 4f, g). Analysis of cell lysates revealed pronounced DQ-collagen I degradation in HA cells, and significantly increased DQ-collagen IV in HN cells (Supplementary Fig. 4h, i). Collagen bands were more fragmented in HN than in HA and HM, indicating a higher degree of ECM processing (Supplementary Fig. 4h).

**Fig. 3:**
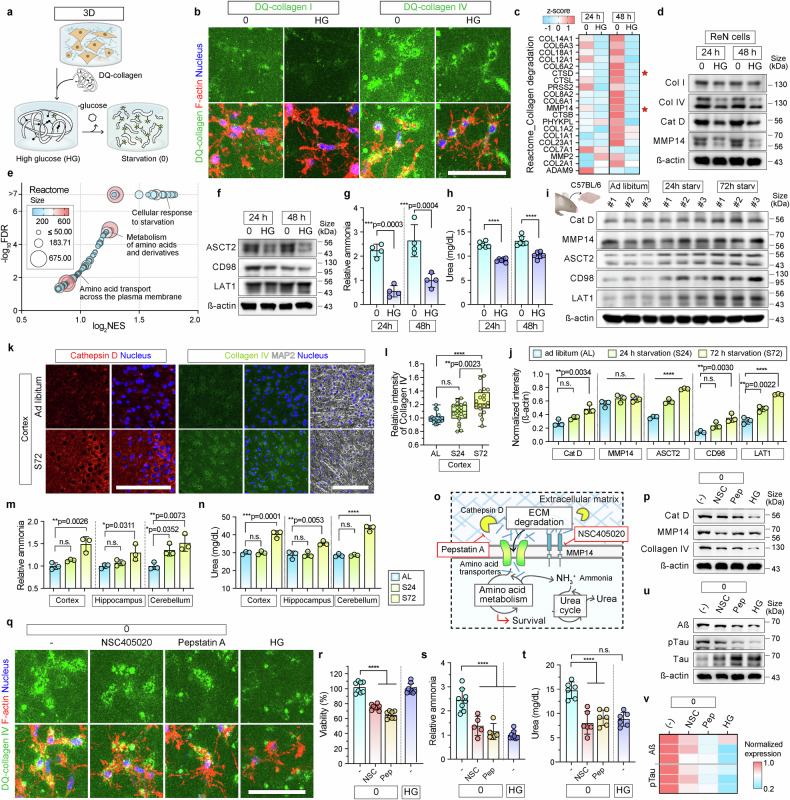
Metabolic reprogramming under hypoglycemia drives ECM degradation and urea production in vitro and in vivo. **a** Schematic illustration of DQ-collagen application to 3D hypoglycemic models. Fluorescence signals emitted by DQ-collagen were increased in 3D hypoglycemic models when substrate ECM components were enzymatically degraded in glucose-free medium. **b** Fluorescence images of degraded collagen types I and IV (green) in HN cultured in 3D hypoglycemic models under starvation (0) and steady-fed (HG) conditions for 24 and 48 h. F-actin (red) and nuclei (blue). Scale bar = 100 µm. **c** Heatmap shows upregulated collagen degradation-related genes from the Reactome pathway in HN without glucose. Data are shown as z-scores. **d** Western blots of intracellular collagen types I and IV, and collagen degradation-related cathepsin D and MMP14, in HN-cultured 3D hypoglycemic models under 0 and HG conditions. β-Actin was used as a loading control. **e** Gene set enrichment analysis of Reactome pathways during starvation. Bubble size indicates gene count, color reflects significance (FDR < 0.05), and the x-axis shows log_2_ NES. **f** Expression of amino acid transporters, ASCT2, CD98, and LAT1 in HN-cultured 3D hypoglycemic models under 0 and HG conditions for 24 and 48 h. β-Actin was used as a loading control. **g**, **h** Analysis of Intracellular ammonia (**g**) and urea (**h**) levels were analyzed under 0 and HG conditions for 24 and 48 h. Data are shown as mean ± SD. Statistical significance was calculated using an unpaired two-tailed t-test (*n* = 4 for (**g**) and *n* = 6 for (**h**); ****p < 0.0001). **i** Expression of cathepsin D, MMP14, ASCT2, CD98, and LAT1 in the cerebral cortex of mice under *ad libitum* (AL) and 24-h (S24) and 72-h (S72) starvation conditions. β-Actin was used as a loading control. **j** Protein expression in the cerebral cortex is shown in (**i**). Graphs represent relative expression of cathepsin D, MMP14, ASCT2, CD98, and LAT1 under all three conditions (*n* = 3 per group; ****p < 0.0001, n.s., not significant). **k** Immunofluorescence staining for cathepsin D (red), collagen type IV (green), and MAP2 (white) in the cerebral cortex under AL and S72. Nuclei are stained blue. Scale bar = 100 µm. **l** Intracellular collagen type IV fluorescence emission in cerebral cortex under AL (*n* = 18), S24 (*n* = 19), and S72 (*n* = 20) conditions. Box and whisker plots respectively represent medians (horizontal bars) and minimum and maximum values at all points (****p < 0.0001). **m**, **n** Intracellular ammonia (**m**) and urea (**n**) concentrations in cerebral cortex, hippocampus, and cerebellum of mice under AL, S24, and S72 (*n* = 3; ****p < 0.0001; n.s., not significant). **o** Inhibition of MT1-MMP and cathepsin D by NSC405020 (NSC) and pepstatin A (Pep), respectively. **p** Intracellular protein expression of cathepsin D, MMP14, and degraded collagen type IV after NSC and Pep treatment under starved conditions compared with those under untreated starved (-) and fed (HG) conditions at 48 h. β-Actin was used as a loading control. **q** Fluorescence images of degraded collagen type IV (green) after NSC and Pep treatment under starvation (0) conditions compared with those under untreated starved (-) and fed (HG) conditions at 48 h. F-actin and nuclei are stained red and blue, respectively. Scale bar = 100 µm. **r** Cell viability in the HN-cultured 3D hypoglycemic model following inhibitor (NSC and Pep) treatment under starved (0) conditions compared with those under untreated starved (-) and fed (HG) conditions after 48 h (*n* = 8; n.s.: not significant; ****p < 0.0001). **s**, **t** Analysis of intracellular ammonia (**s**) and urea (**t**) after NSC and Pep treatment under starved (0) conditions compared with those under untreated starved (-) and fed (HG) conditions after 48 h (*n* = 8 in untreated and *n* = 5 in inhibitor-treated groups in (**s**), *n* = 6 in (**t**); ****p < 0.0001). Scatter dot plots in (**j**), (**m**), (**n**), (**r**), (**s**), and (**t**) represent means ± SD with bars and error bars. Statistical significance in (**j**), (**m**), (**n**), (**r**), (**s**), and (**t**) was calculated using ordinary ANOVA, followed by Tukey multiple comparison tests. **u** Protein expression of the Aß, pTau, Tau, S100B, vimentin, and iNOS after NSC and Pep treatment under starved (0) conditions compared with those under the untreated starved (-) and fed (HG) conditions after 48 h. ß-actin was used as a loading control. **v** Heatmap showing relative expression values of the neurodegenerative markers (Aß and pTau/Tau ratio) after NSC and Pep treatment, based on the Supplementary Fig. 6c. ECM extracellular matrix, FDR false discovery rate, HN human neural cell, NES normalized enrichment score

We visualized the degradation of DQ-collagen type IV using fluorescence imaging. The intensity of fluorescence emission was significantly higher for degraded DQ-collagen type IV than for type I in HN cells under glucose-starved conditions (Supplementary Fig. 4j, k). In contrast, HA exhibited mesh-like fluorescence of DQ-collagen without significant changes in intensity, whereas minimal signal HM signals indicated limited ECM degradation activity. These findings were further validated using a 3D hypoglycemic model, wherein DQ-collagen I and IV were co-incorporated into the ECM (Fig. 3a). The fluorescent signal was more intense from DQ-collagen type IV than type I in HN cultured in the 3D model (Fig. 3b). This supported the notion that neurons preferentially use collagen type IV under hypoglycemia.

These findings overall showed that human neurons actively degrade and metabolize ECM components, particularly type IV collagen, to survive glucose deprivation. Conversely, ECM utilization appears to be limited or less effective in astrocytes and microglia under glucose-deprived conditions.

### Activated amino acid metabolism increases ammonia and urea production in glucose-starved neurons

Genes involved in collagen degradation were significantly upregulated in glucose-starved neurons at 24 and 48 h (Fig. 3c, Supplementary Fig. 5a). Among these, transcriptional upregulation was the most prominent for cathepsin D (CTSD) and membrane type 1 matrix metalloproteinases (MT1-MMP or MMP14) (Supplementary Fig. 5a). The levels of CTSD and MMP14 proteins were substantially elevated under glucose starvation and coincided with increased intracellular levels of collagen types I and IV. These results suggested active ECM degradation and uptake (Fig. 3d).

A transcriptomic analysis of the Reactome database further revealed significantly enriched gene sets associated with amino acid metabolism and transport, particularly “metabolism of amino acids and derivatives”, “amino acid transport across the plasma membrane”, and “cellular response to starvation” (Fig. 3e, Supplementary Fig. 5b–g). The gene sets “protein processing in the endoplasmic reticulum”, “phagosome”, “autophagy”, and “lysosome” from the KEGG pathway were also notably upregulated under glucose deprivation (Supplementary Fig. 5h). Most genes in the “metabolism of amino acids and derivatives” category were consistently upregulated at 24 and 48 h of glucose starvation (Supplementary Fig. 5d, e).

Among the upregulated genes encoding amino acid transporters, the expression of *SLC1A5* (sodium-dependent neutral amino acid transporter 2, ASCT2), *SLC3A2* (activator of dibasic and neutral amino acid transporter, 4F2 or CD98), and *SLC7A5* (large neutral amino acid transporter 1, LAT1) was substantially increased under glucose starvation (Supplementary Fig. 5d–g). These findings were validated at the protein level, which revealed significant upregulation of ASCT2, CD98, and LAT1 in neurons under glucose starvation (Fig. 3f, Supplementary Fig. 6a).

We further assessed the metabolic consequences of amino acid catabolism by evaluating intracellular levels of ammonia/ammonium (NH_3_/NH_4_^+^) and urea in the 3D hypoglycemic models. Both were significantly elevated under glucose starvation, indicating enhanced amino acid metabolism and activation of the urea cycle (Fig. 3g, h, respectively). This metabolic shift was also found in vivo (Fig. 3i–n). The expression of cathepsin D and amino acid transporters (ASCT2, LAT1, and CD98) was significantly elevated in the cerebral cortex of mice that were starved for 72 h (Fig. 3i–k). Internalized type IV collagen levels were also significantly elevated (Fig. 3k, l), and levels of NH_3_/NH_4_^+^ and urea were significantly increased in the cerebral cortex, hippocampus, and cerebellum in glucose-starved mice (Fig. 3m, n).

Collectively, these findings confirmed that neurons use the ECM, particularly collagen type IV, as an alternative energy source during hypoglycemic stress. Degraded ECM components are internalized by amino acid transporters (SLC family), thereby facilitating amino acid metabolism, which leads to the production of ammonia/ammonium and urea both in vitro and in vivo.

### ECM degradation triggers neurodegenerative processes in glucose-starved neurons

Based on our previous findings, we speculated that ECM degradation directly contributes to neurodegenerative processes in neurons. We therefore examined the expression of neurodegenerative markers in HN, HA, and HM cells incubated with DQ-collagen I and IV under 2D cultures glucose-starved conditions (Supplementary Fig. 7). Expression of the neurodegenerative and neuroinflammatory markers, including Aß, tau, pTau, and iNOS, was significantly elevated in HN cells incubated with DQ-collagen I and IV (Supplementary Fig. 7a, b). However, no significant changes were observed in the expression of reactive astrocyte markers (GFAP, vimentin, and iNOS) (Supplementary Fig. 7c, d) or microglial activation markers (Iba1, CD11b, and CD40) under the same conditions (Supplementary Fig. 7e, f). These findings are consistent with the observations made in the 3D hypoglycemic model (Fig. 2, Supplementary Fig. 2), supporting the notion that ECM degradation plays a specific and direct role in neuronal, but not glial, neurodegeneration during glucose deprivation.

To confirm that ECM degradation drives neurodegeneration, we inhibited two key ECM-degrading proteases, cathepsin D and MMP14, using pepstatin A (Pep) and NSC405020 (NSC), respectively (Fig. 3o). Treatment with these inhibitors under glucose-starved conditions effectively suppressed cathepsin D and MMP14 expression (Fig. 3p, Supplementary Fig. 6b). Consequently, the degradation of collagen IV was markedly reduced (Fig. 3q), and intracellular levels of collagen type IV were also decreased (Fig. 3p, Supplementary Fig. 6b).

Although protease inhibition led to decreased ECM utilization, it also significantly reduced neuronal cell viability under glucose starvation (Fig. 3r), which was consistent with the role of ECM-derived as an alternative energy source. Moreover, intracellular levels of ammonia and urea were significantly decreased after Pep and NSC treatment (Fig. 3s, t), indicating the suppression of amino acid metabolism. Notably, the expression of AD-related pathogenic markers, including Aβ, tau, and pTau, was significantly reduced in Pep- and NSC-treated cells (Fig. 3u, v, Supplementary Fig. 6c). These findings suggest that blocking ECM degradation not only impairs metabolic compensation but also mitigates hypoglycemia-induced neurodegeneration. These results highlight the dual role of ECM degradation in supporting neuronal survival through metabolic adaptation, while simultaneously promoting neurodegenerative processes under hypoglycemic stress.

### Hypoglycemia triggers neurodegeneration mediated by SRF/MRTF-A

**Fig. 4:**
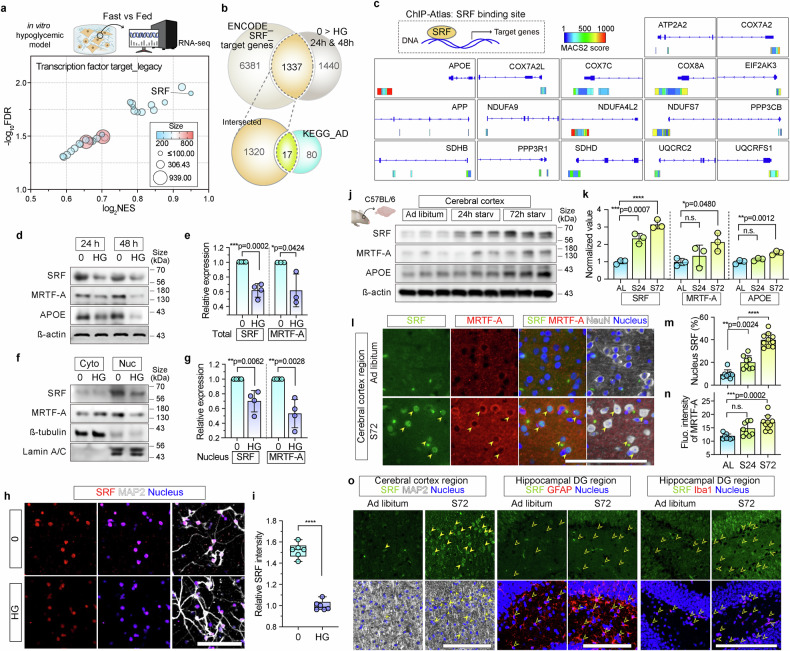
Activation of SRF and MRTF-A regulates neurodegeneration under hypoglycemia. **a** Gene set enrichment analysis using the TFT Legacy collection from the Molecular Signatures Database (MSigDB) identified SRF as significantly enriched in the starvation group versus the fed (HG) group after 48 h. The bubble plot represents significantly enriched gene sets with a false discovery rate (FDR) < 0.05 and the normalized enrichment score (NES) as a log_2_ value. **b** A Venn diagram represents the intersected genes. Top: the intersection between SRF target genes (ENCODE dataset) and genes upregulated in the starvation (0) versus the HG group. Bottom: Overlap between these intersected genes and Alzheimer’s disease (AD) pathway genes (KEGG pathway AD: hsa05010). **c** ChIP-Atlas analysis showing SRF binding to 17 AD-related genes in the model-based analysis of ChIP-seq version 2 (MACS2) scores over 50. The intensity of gene‒protein binding is represented by the MACS2 score. **d**, **f** Western blot analysis of SRF, MRTF-A, and APOE (**d**), and SRF/MRTF-A in nuclear and cytoplasmic fractions of (**f**) 3D-cultured HN under 0 and HG conditions for 24 and 48 h. β-Actin was used as a loading control for total proteins. β-Tubulin and Lamin A/C were loading controls for cytoplasmic and nuclear fractions, respectively. **e**, **g** Quantification of total (**e**) and nucleic (**g**) SRF and MRTF-A expression under the 0 and HG conditions. Graphs represented the relative expression values by comparing starved conditions (*n* = 3). **h**, **i** Immunofluorescence staining images (**h**) of MAP2 (white) and SRF (red) without (0) and with (HG) glucose. Nuclei were stained with DAPI (blue). Scale bar = 100 µm. The fluorescence intensity of SRF (**i**) represented the relative expression value, as determined by comparing the HG conditions (*n* = 5, *p* < 0.0001). **j** Protein expression of SRF, MRTF-A, and APOE in the cerebral cortex of mouse brains under AL, S24, and S72 conditions. β-Actin was used as a loading control. **k** Quantitative expression values of SRF, MRTF-A, and APOE protein expression compared with those in control AL conditions in the cerebral cortex of mouse brains. Graphs represented the relative expression value by comparing AL conditions (*n* = 3; ****p < 0.0001, n.s., not significant). **l** Immunofluorescence staining of SRF (green) and MRTF-A (red) under AL and S72 conditions in the cerebral cortex of mouse brains. NeuN (white) and nuclei (blue) were stained. Scale bar = 100 µm. **m**, **n** Image-based quantification of nuclear SRF-positive cells (**m**) and MRTF-A fluorescent intensity (**n**) in the cerebral cortex regions under AL, S24, and S72 conditions. **o** Immunofluorescence staining of SRF (green) with the neuronal (MAP2, white), astrocyte (GFAP, red), and microglia marker (Iba1, red) in the cerebral cortex and hippocampal dentate gyrus (DG) regions of mice brains under AL and S72 conditions. Nuclei were stained with DAPI (blue). Full yellow arrows represented neurons with nucleus SRF, and empty yellow arrows represented astrocytes and microglia lacking SRF expression under AL and S72 conditions in the cerebral cortex and hippocampus DG regions in starved conditions. Scale bar = 100 µm. Scatter dot plots in (**e**), (**g**), (**k**), (**m**), and (**n**) represent the mean ± SD. Box and whisker plots in (**i**) represent the median (horizontal bars) and minimum and maximum values with all points, respectively. Statistical significance was determined using an unpaired two-tailed t-test. Statistical significance in (**e**), (**g**), and (**i**) was calculated using an unpaired two-tailed t-test. Statistical significance in (**k**), (**m**), and (**n**) was calculated using an ordinary one-way analysis of variance, followed by the Tukey multiple comparison test. HN human neural cell, TFT transcription factor targets

To elucidate the molecular mechanism underlying neurodegeneration under hypoglycemic conditions, we attempted to identify the transcriptional regulators activated by glucose starvation. We screened targets of transcription factors using GSEA of transcriptomic data from 3D hypoglycemic models *via* the TFT_Legacy (legacy transcription factor targets) collection. SRF was consistently and significantly enriched by glucose starvation at 24 and 48 h (Supplementary Fig. 8a, b for 24 h; Fig. 4a, Supplementary Fig. 8c for 48 h), suggesting its involvement in the cellular responses to hypoglycemia.

Further analysis using the online chromatin immunoprecipitation sequencing (ChIP-seq) database, ChIP-Atlas,^31^ revealed that SRF binds to several genes associated with AD (Fig. 4b, c). Among 7718 SRF target genes in the Encyclopedia of DNA Elements (ENCODE) dataset, 1337 were significantly upregulated under starvation (0) (Fig. 4b, top). Among these, KEGG analysis associated 17 genes with AD (Fig. 4b, bottom). The results of the ChIP-Atlas analysis indicated that SRF has the potential to bind to 17 AD-related genes, including apolipoprotein E (APOE), which is a major risk factor for AD^32^ (Fig. 4b, c, Supplementary Fig. 9a). Furthermore, SRF was also predicted to bind to the promoters of genes involved in ECM degradation (specifically *CTSD* and *MMP14*) and amino acid transport (*SLC7A5*, *SLC1A5*, and *SLC3A2*) (Supplementary Fig. 9b). These findings indicate that SRF may coordinates neurodegenerative signaling and ECM utilization under hypoglycemic stress.

To validate this regulatory axis, we examined the expression of SRF and its coactivator, MRTF.^33^ Analysis of the human AD dataset (GSE63063) revealed that *SRF* (SRF) and *MKL1* (MRTF-A) were significantly upregulated in the brains of patients with mild cognitive impairment (MCI) and AD, whereas *MKL2* (MRTF-B) was not (Supplementary Fig. 8d). Therefore, we subsequently focused on MRTF-A. Total and nuclear levels of SRF and MRTF-A in our 3D hypoglycemic model were significantly increased (Fig. 4d–i), and APOE protein levels were upregulated (Fig. 4d) under glucose starvation.

Similarly, MRTF-A expression was significantly elevated in HN cells incubated with DQ-collagen in 2D models under hypoglycemic conditions, whereas minimal or no induction was evident in HA and HM (Supplementary Fig. 10a–c). Basal levels of SRF and MRTF-A were low in the 2D-cultured HA, and HM was not significantly altered under glucose starvation or DQ-collagen treatment (Supplementary Fig. 10a–c).

The ECM composition further affected the nuclear localization of SRF and MRTF-A in HN cells. DQ-collagen IV treatment led to a significant increase in nuclear SRF, whereas DQ-collagen I exhibited a non-significant increase in nuclear MRTF-A (Supplementary Fig. 10d–f). In contrast, under HG conditions, both nuclear SRF and MRTF-A levels remained low, with no notable changes following DQ-collagen treatment (Supplementary Fig. 10d–h).

Collectively, these findings suggest that SRF/MRTF-A acts as a crucial regulatory hub to mediate both metabolic adaptation and transcription of neurodegeneration-associated genes in neuronal pathology, specifically in neurons.

### SRF/MRTF-A is activated in the hypoglycemic mouse brain

To validate the activation of SRF/MRTF-A signaling in vivo, we examined the brains of starved mice after 72 h of glucose deprivation. The protein expression levels of SRF, MRTF-A, and APOE were markedly upregulated in the cerebral cortex (Fig. 4j–n, Supplementary Fig. 11c–e). Moreover, the expression of MRTF-A and APOE was increased in the hippocampus, whereas that of SRF was elevated in the cerebellum (Supplementary Fig. 11). These results further substantiated that activation of this signaling pathway in the brain is regiospecific. Immunofluorescence analysis revealed a significant increase in the number of SRF-positive neuronal nuclei in the cerebral cortex, where MRTF-A was co-expressed with SRF under starvation (Fig. 4l–n). The expression of SRF was predominantly localized in neurons with the neuronal marker MAP2, but not with the astrocyte marker GFAP or the microglial marker Iba1, in the cerebral cortex and hippocampal dentate gyrus (DG) region (Fig. 4o).

These in vivo results were consistent with those obtained from the 3D models in vitro and confirmed that SRF and MRTF-A are selectively activated in neurons under hypoglycemic stress. Collectively, these findings demonstrate that SRF/MRTF-A is a central mediator of the neuronal response to glucose deprivation, contributing to hypoglycemia-induced neurodegeneration.

### Inhibition of SRF/MRTF-A attenuates hypoglycemia-induced neurodegeneration

**Fig. 5:**
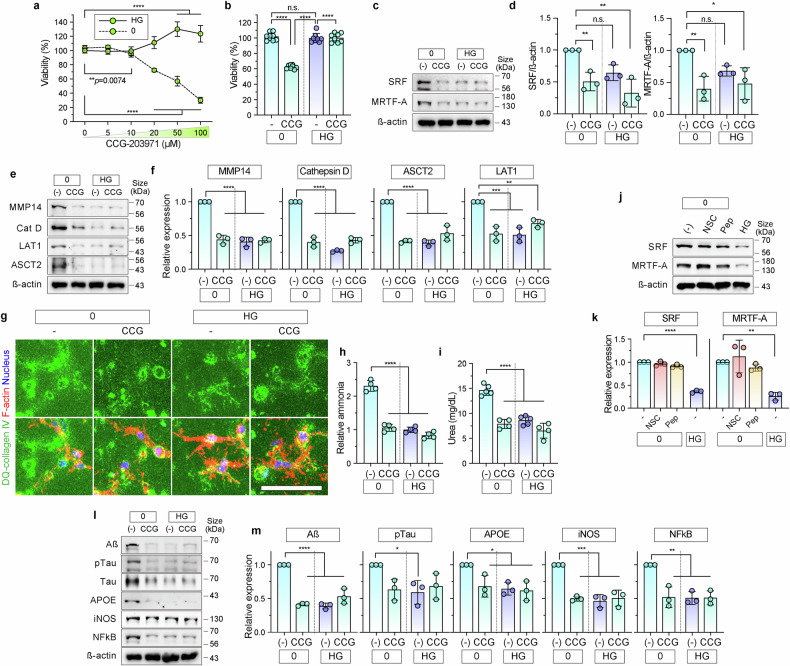
Inhibition of SRF and MRTF-A using CCG-203971. **a**, **b** Cell viability in the 3D-cultured HN after a dose-dependent treatment of CCG-203971 (CCG) under starved (0) and fed (HG) conditions. Bar graph (**b**) shows cell viability at 20 µM CCG (*n* = 8, ****p < 0.0001). **c**, **d** Protein expression of SRF and MRTF-A in HNs in 3D hypoglycemic model after 48 h incubation with 20 µM CCG under 0 and HG conditions. β-Actin was used as a loading control. Relative expression of SRF and MRTF-A (**d**) in untreated starved (-) and HG conditions (*n* = 3; n.s.). **e**, **f** Protein expression of protease (MMP14 and cathepsin D) and amino acid transporters (LAT1 and ASCT2) after CCG treatment under 0 and HG conditions. β-Actin was used as a loading control. Graphs (**f**) show the relative expression level by comparing the untreated starved (-) control with HG conditions (*n* = 3; n.s., not significant). **g** Fluorescence images of degraded collagen type IV (green) after CCG treatment under starvation (0) conditions compared with those of untreated starved (-) and fed (HG) conditions for 48 h. F-actin and nuclei are stained red and blue, respectively. Scale bar = 100 µm. **h**, **i** Analysis of intracellular ammonia (**h**) and urea (**i**) after CCG treatment under starvation (0) conditions compared with that in the untreated (-) starvation and fed (HG) conditions after 48 h (**h**, *n* = 4; **I**, *n* = 4–5; ****p < 0.0001). **j**, **k** Protein expression levels of SRF and MRTF-A after NSC and Pep treatment under starvation (0) conditions compared with those in the untreated fed (HG) conditions. β-Actin was used as a loading control. Quantification of SRF and MRTF-A expression (**k**) compared with those in untreated starved (-) and HG conditions. **l**, **m** Protein expression of Aß, pTau, Tau, APOE, iNOS, and NFkB in the HN-cultured hypoglycemic model after CCG treatment under both 0 and HG conditions for 48 h. β-Actin was used as a loading control. Quantification of relative protein expression (**m**) in untreated starved (-) and HG conditions (*n* = 3). Data in scatter dot plots (**b**), (**d**), (**f**), (**h**), (**k**), and (**m**) are shown as means ± SD with bars and error bars. Significance was calculated using ordinary ANOVA, followed by Tukey multiple comparison tests. HN human neural cell

We added the SRF/MRTF pathway inhibitor CCG-203971 (CCG) to the 3D hypoglycemic model to evaluate the functional role of SRF/MRTF-A in ECM utilization and AD-like neurodegeneration. The inhibitor (0–100 µM) dose-dependently decreased neuronal cell viability (Fig. 5a), which remained stable in the presence of glucose, indicating that SRF/MRTF-A plays a crucial role in neuronal survival under glucose deprivation. At 20 µM, CCG reduced viability by approximately 40% in the starved (0) conditions, while no substantial differences were observed in HG conditions (Fig. 5b). The expression of SRF and MRTF-A was substantially reduced by CCG under glucose starvation (Fig. 5c, d), thereby inhibiting this transcriptional axis.

Consistent with the role of SRF/MRTF-A in ECM remodeling, levels of the proteases cathepsin D and MMP14, as well as the amino acid transporters ASCT2 and LAT1, were significantly reduced by CCG under glucose starvation (Fig. 5e, f). Assays of DQ-collagen showed that CCG decreased ECM degradation (Fig. 5g). Furthermore, intracellular levels of ammonia and urea, which are byproducts of amino acid catabolism, were significantly decreased in neurons exposed to CCG (Fig. 5h, i), indicating that downstream metabolic reprogramming was inhibited.

We aimed to clarify signaling involved in hypoglycemic adaptation by evaluating changes in SRF and MRTF-A expression caused by the protease inhibitors (NSC and Pep, Fig. 5j, k) and time-dependent starvation between 0 and 48 h (Supplementary Fig. 12). We confirmed that there were no significant differences in the expression of SRF and MRTF-A in the NSC- and Pep-treated cells under the starved condition (Fig. 5j, k). We also verified that the expression of nuclear and total SRF and MRTF-A was significantly increased during short-term starvation for 12–24 h, whereas cathepsin D and MMP14 expression was elevated during long-term starvation for 48 h (Supplementary Fig. 12a–d). Based on these results, we concluded that SRF and MRTF-A are initially activated in response to glucose depletion and function as transcription factors for proteases involved in metabolic reprogramming (Supplementary Fig. 12e).

Importantly, CCG mitigated the key molecular hallmarks of AD. After SRF-MRTF-A inhibition, expression of the neurodegenerative markers—Aβ, pTau, and APOE—as well as the neuroinflammatory marker nuclear factor kappa-light-chain-enhancer of activated B cells (NFκB) and iNOS, was significantly reduced under glucose depletion (Fig. 5l, m).

Collectively, these findings showed that SRF/MRTF-A plays a dual role in promoting neuronal survival *via* ECM-derived metabolic compensation, while concurrently facilitating amyloidogenic and neurodegenerative pathways under hypoglycemic stress. Therefore, pharmacological inhibition of SRF and MRTF-A presents a promising therapeutic approach for preventing hypoglycemia-induced neurodegeneration and AD-like pathology.

### Urea induces neurodegenerative pathology

**Fig. 6:**
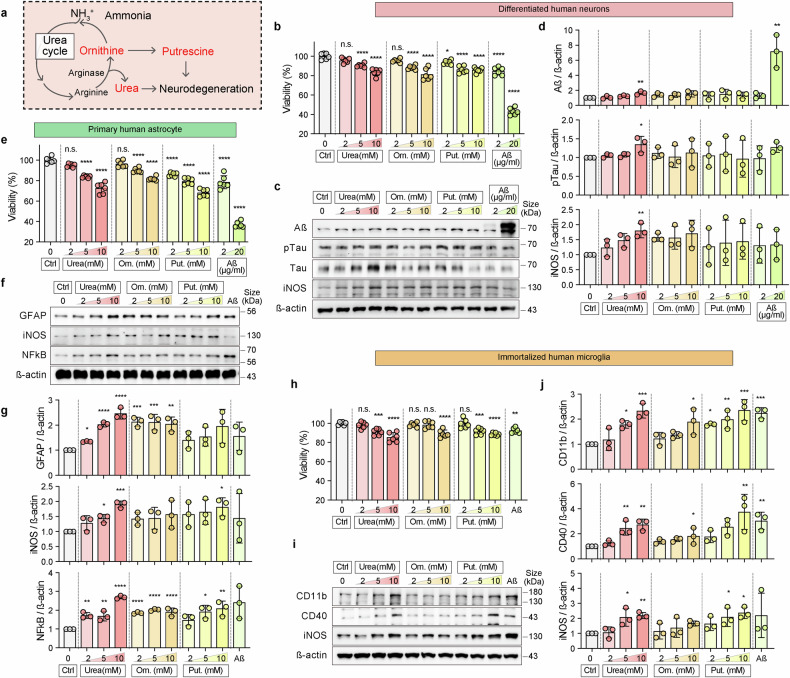
Urea cycle byproducts induce neurodegeneration in 3D-hypoglycemic in vitro models. **a** Ammonia is metabolized through the urea cycle to urea and its by-products, including ornithine (Orn) and putrescine (Put). To assess their potential neurotoxicity, urea, Orn, and Put were treated in the 3D-hypoglycemic models for 48 h. All treatments were performed in the glucose-containing medium, and compared with amyloid beta (Aß) treatment. **b**, **e**, **h** Comparison of HN (**b**), HA (**e**), and HM (**h**) viability in 3D-models incubated with urea, Orn, Put, and Aß for 48 h. Cell viability was normalized to the untreated control (set as 100%). The scatter dot plot represents the mean ± SD with bars and error bars. Statistical significance was calculated using an ordinary one-way analysis of variance, followed by Tukey’s multiple comparison tests (*n* = 6; ****p < 0.0001, n.s., not significant). **c**, **f**, **i** Protein expression of neurodegenerative and neuroinflammatory markers (**c** Aß, pTau, Tau, and iNOS), reactive astrocyte markers (**f** GFAP, iNOS, and NFκB), and microglial activation markers (**i** CD11b, CD40, and iNOS) under urea-, Orn-, Put-, and Aß-treated conditions for 48 h in 3D-cultured HN (**c**), HA (**f**), and HM (**i**) models. β-Actin was used as a loading control. **d**, **g**, **j** Quantification of protein expression levels from (**c**), (**f**), and (**i**), normalized to untreated control. Graphs represented the relative expression values of neurodegenerative markers (**d** Aß, ratio of pTau/Tau, and iNOS), reactive astrocyte markers (**g** GFAP, iNOS, and NFκB), and microglial activation markers (**j** CD11b, CD40, and iNOS) under urea-, Orn-, Put-, and Aß-treated conditions for 48 h in 3D-cultured HN (**d**), HA (**g**), and HM (**j**) models. The scatter dot plot represents the mean ± SD. Statistical significance was calculated using an ordinary one-way analysis of variance, followed by Tukey multiple comparison tests (*n* = 3; *p < 0.05, **p < 0.01, ***p < 0.001, ****p < 0.0001). HA human astrocyte, HM human immortalized microglia, HN human neural cell

Given that urea levels are elevated in neurodegenerative diseases, including AD,^34,35^ we hypothesized that hypoglycemia-induced urea acts as a neurotoxic factor that contributes to the progression of neurodegeneration. To elucidate the urea-induced neurodegenerative mechanism, we performed a dose-dependent study using urea in a 3D hypoglycemic model. We also examined the urea cycle derivatives, ornithine (Orn) and putrescine (Put)^36^ (Fig. 6a), and compared their effects to the Aß-treated condition. To determine the concentration of urea cycle derivatives, we conducted a concentration-dependent study using urea, Orn, and Put in HN-cultured 3D models (Supplementary Fig. 13). We treated the cells with urea at concentrations ranging from 0 to 200 mM and Orn and Put at concentrations ranging from 0 to 100 mM. Cell viability remained above 70% even at 200 mM urea, whereas viability conspicuously declined when incubated with exceeding 10 mM Orn and Put. We then compared the effects at 2, 5, and 10 mM each urea, Orn, and Put for accuracy.

All three compounds dose-dependently diminished neuronal cell viability, similar to Aß-induced neurotoxicity (Fig. 6b). Furthermore, expression of the neurodegenerative markers Aß, pTau, and the neuroinflammatory marker iNOS was elevated in the presence of urea (Fig. 6c, d). However, no significant differences were observed between Orn and Put treatments.

Markers indicating reactive astrocytes were upregulated in the 3D differentiated system, whereas HA exhibited no alteration in astrocytic markers after glucose deprivation. Hence, we speculated that urea produced in neurons activates glial cells. As we expected, primary human astrocytes demonstrated a significant decline in viability following treatment with urea, Orn, Put, and Aß (Fig. 6e). Urea markedly affected the upregulation of the reactive astrocyte markers,^37^ GFAP, iNOS, and NFκB, in a dose-dependent manner. Urea and Orn (both 10 mM) elevated levels of GFAP by approximately 2.5- and 2.0-fold, respectively, and iNOS by approximately 1.9- and 1.8-fold, respectively. Urea-, Orn-, and Put also increased NFκB levels by approximately 2.7-, 1.9-, and 2.1-fold, respectively (Fig. 6f, g). Microglial viability also notably decreased following these treatments (Fig. 6h). In particular, urea increased CD11b, CD40, and iNOS levels by approximately 2.6-, 2.7-, and 2.2-fold, respectively, whereas Put increased CD11b, CD40, and iNOS by approximately 2.4-, 2.6-, 2.4-fold, respectively (Fig. 6i, j).

These results suggest that downstream metabolic byproducts, particularly urea and its derivatives, can drive astrocytic and microglial activation, which is consistent with the in vivo responses of the starved mouse brain (Fig. 1). Glucose starvation alone did not significantly alter glial reactivity in vitro (Fig. 2, Supplementary Fig. 2). However, we verified that urea accumulation is a key mediator of hypoglycemia-induced glial cell activation and progression of neurodegeneration.

### Glucose reversed neurodegenerative features and reduced levels of urea

**Fig. 7:**
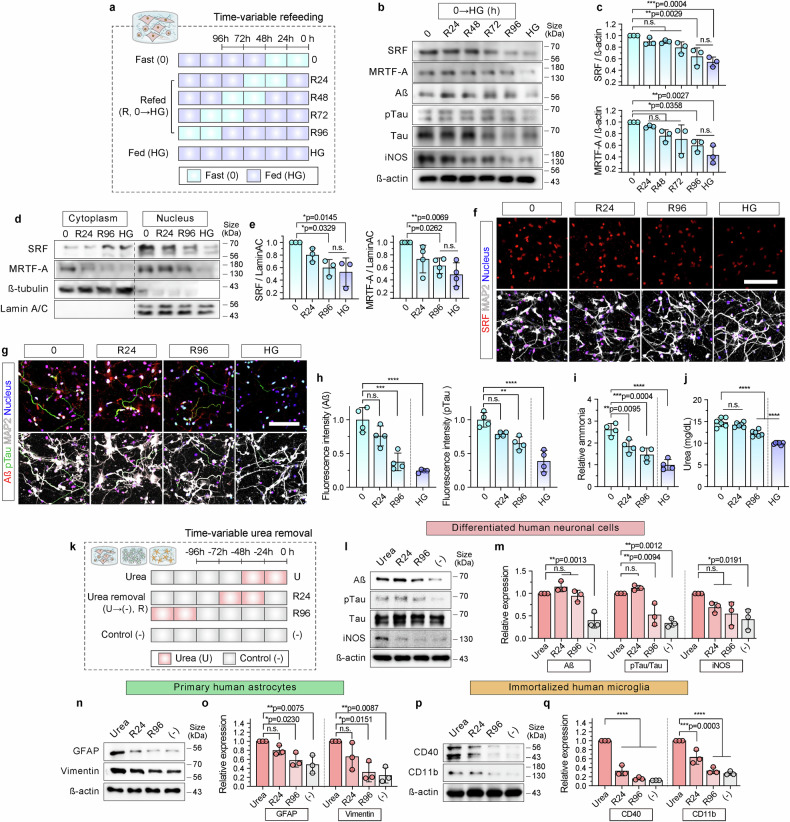
Reversal of AD-like pathogenesis via glucose refeeding and urea removal in 3D hypoglycemic models. **a** Experimental timeline for the time-dependent replacement with glucose (0 = 0 mM; HG = 30 mM glucose). After 48 h without glucose starvation, cells were incubated for 24–96 h with medium containing glucose (HG). **b** Expression of SRF, MRTF-A, Aβ, pTau, Tau, and iNOS proteins in HN in 3D hypoglycemic models following glucose refeeding (HG→0) for 24 to 96 h, compared with 0 and HG conditions. β-Actin was used as a loading control. **c** Quantification of total SRF and MRTF-A protein levels from (**b**) relative to starved conditions. Graphs represented the relative expression values by comparing starved conditions (*n* = 3; n.s.; not significant). **d** Western blot of SRF and MRTF-A in cytoplasmic and nuclear fractions under glucose refeeding conditions (HG→0) for 24 and 96 h compared with the starved (0) and HG conditions. β-Tubulin and Lamin A/C were used as loading controls for the cytoplasm and nucleus, respectively. **e** Quantification of nuclear SRF and MRTF-A expression from (**d**). Graphs represented the relative expression values by comparing starved (0) conditions (*n* = 3). **f**, **g** Immunofluorescence staining of SRF (red) (**f**) and neurodegenerative markers (**g** Aβ [red] and pTau [green]) after 24 and 96 h of glucose refeeding compared with those in the 0 and HG conditions. MAP2 (white) and nuclei (blue) were stained. Scale bar = 100 µm. **h** Fluorescence intensity (**h**) of Aß and pTau of glucose refeeding compared with those of the 0 and HG conditions (*n* = 4). **i**, **j** Analysis of intracellular ammonia (**i**) and urea (**j**) after 24 and 96 h of glucose refeeding compared to the 0 and HG conditions (*n* = 4 in (**i**), *n* = 6 in (**j**); ****p < 0.0001, n.s., not significant). **k** Experimental timeline for urea removal (R, U = 100 mM urea; (-) = urea-free medium). After 48 h of urea treatment, the medium was changed to urea-free media for 24 (R24) to 96 h (R96). All experiments were performed in glucose-containing medium. **l**, **n**, **p** Expression of neurodegenerative markers (**l** Aß, pTau, Tau, and iNOS), reactive astrocyte markers (**n** GFAP and vimentin), and microglial activation markers (**p** CD11b and CD40) after urea removal for 24 (R24) and 96 h (R96) in 3D cultured HN (**l**), HA (**n**), and HM (**p**) models. ß-actin was used as a loading control. **m**, **o**, **q** Quantitative protein expression in (**l**), (**n**), and (**p**). Values of the neurodegenerative markers (**m** Aß, pTau, Tau, and iNOS), reactive astrocyte markers (**o** GFAP and vimentin), and microglial activation markers (**q** CD11b and CD40) proteins after the time-variable urea removal for R24 and R96 in 3D-cultured HN (**m**), HA (**o**), and HM (**q**) models. Graphs represented the relative expression values by comparing urea-treated conditions (*n* = 3; n.s. not significant; ****p < 0.0001). The scatter dot plots in (**c**), (**e**), (**h**), (**I**), (**j**), (**m**), (**o**), and (**q**) represent the mean ± SD. Statistical significance was calculated using an ordinary one-way analysis of variance, followed by Tukey’s multiple comparison test. HA human astrocyte, HM human immortalized microglia, HN human neural cell

To investigate whether the neurodegenerative features induced by hypoglycemia were reversible, we performed time-course experiments of glucose refeeding (0→HG) for 24, 72, and 96 h in a 3D hypoglycemic model (Fig. 7a). Glucose refeeding at these time points progressively reduced SRF and MRTF-A expression, with significant downregulation at 96 h (Fig. 7b, c, f). Correspondingly, the nuclear localization of SRF and MRTF-A also progressively decreased over time (Fig. 7d, e, f). This decrease was accompanied by a decline in the neurodegenerative markers Aß and pTau, the neuroinflammatory marker iNOS (Fig. 7b, g, h), and intracellular ammonia and urea levels (Fig. 7i, j), further linking urea metabolism to the neurodegenerative process.

To directly examine the role of urea clearance in cellular recovery, we exposed the cells to urea for 48 h and then switched to a urea-free medium for either 24 or 96 h (Fig. 7k). The pTau/Tau ratio was significantly reduced after 96 h of urea withdrawal, whereas Aß and iNOS levels remained largely unchanged (Fig. 7l, m). Astrocytic reactivity also declined, with significant downregulation of vimentin and GFAP after 96 h (Fig. 7n, o). Levels of the microglial activation markers CD40 and CD11b decreased within 24 h of urea withdrawal (Fig. 7p, q).

These findings indicated that either glucose or direct urea withdrawal attenuates the neurodegenerative effects of hypoglycemia. We suggest that the nuclear localization of SRF/MRTF-A reflects a transient type of response because some SRF/MRTF-A remained in nuclei even after glucose was restored (Fig. 7d, e), potentially sustaining low-level transcription of stress-responsive genes.

### Refeeding attenuates neurodegenerative features in mouse brains

**Fig. 8:**
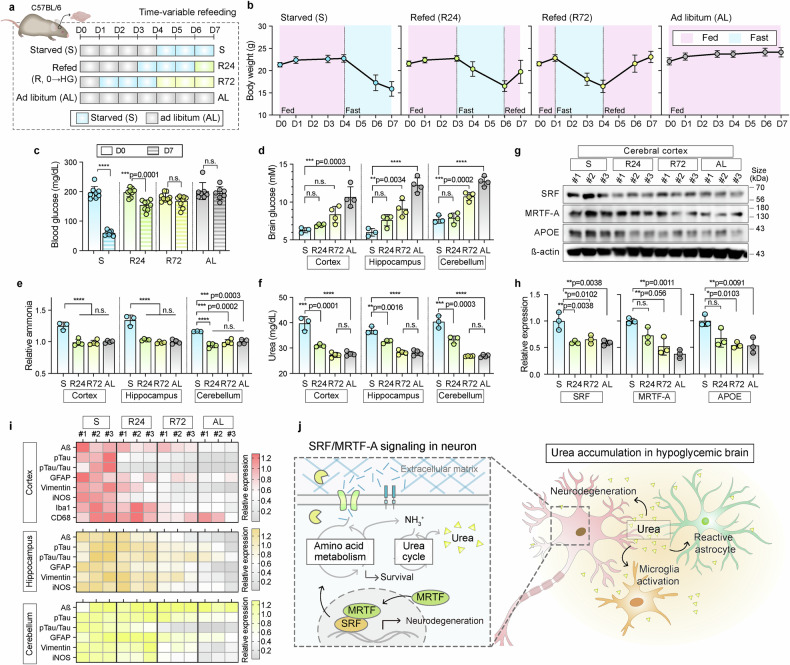
Refeeding restores glucose homeostasis and attenuates AD-like pathogenesis via SRF/MRTF-A regulation in hypoglycemic mouse brains. **a** Experimental timeline for time-variable refeeding (S starvation; AL ad libitum). After 72 h of starvation, the mice were refed for 24 (R24) to 72 h (R72). **b** Analysis of body weight changes in each condition, (i) S, (ii) R24, (iii) R72, and (iv) AL. Body weight was measured under all experimental conditions (*n* = 7–9). **c** Analysis of blood glucose level under the time-variable refeeding. Blood glucose level measured before and after the experiments (*n* = 7–9; ****p < 0.0001, n.s., not significant). **d**–**f** Analysis of intracellular glucose (**d**), ammonia (**e**), and urea (**f**) levels in the cerebral cortex, hippocampus, and cerebellum after refeeding. Intracellular glucose (**d**), ammonia (**e**), and urea (**f**) levels were measured after the refeeding experiment (*n* = 3 to 4; ****p < 0.0001, n.s., not significant). **g** Western blot analysis of the SRF, MRTF-A, and APOE proteins in the cerebral cortex of mouse brains after time-variable refeeding for R24 to R72 compared to the S and AL conditions. ß-actin was used as a loading control. **h** Quantification of SRF, MRTF-A, and APOE protein levels in the cerebral cortex regions of mice brains after time-variable refeeding for R24 to R72. Graphs represented the relative expression values by comparing starved conditions (*n* = 3; n.s., not significant). The scatter dot plots in (**c**), (**d**), (**e**), (**f**), and (**h**) represent the mean ± SD. Statistical significance was calculated using an ordinary one-way analysis of variance, followed by Tukey’s multiple comparison tests. **i** Heatmap showing relative expression of the neurodegenerative markers (Aß, pTau, pTau/Tau ratio, and iNOS), reactive astrocyte markers (GFAP and vimentin), and microglial activation markers (Iba1 and CD68) proteins in the cerebral cortex, hippocampus, and cerebellum regions of mouse brains. The heatmap was generated based on the relative expression value from Supplementary Fig. 14. **j** Schematic illustration of the neuronal response to hypoglycemia. Under glucose deprivation, neurons activate SRF/MRTF-A-mediated metabolic reprogramming to support survival by degrading extracellular matrix, which leads to urea production and Alzheimer’s disease (AD)-like pathology. The excess urea and its derivatives further promote the activation of astrocytes and microglia. Refeeding reverses their effects by deactivating SRF/MRTF-A and reducing urea levels. A schematic illustration was created using Adobe Illustrator

To assess whether glucose refeeding reversed AD-like pathology in vivo, we performed time-course refeeding experiments in hypoglycemic mice for 24 and 72 h (Fig. 8a). We found that body weight gradually increased, reaching 23.1 g after 72 h of refeeding, compared with 15.9 g under starvation (Fig. 8b). Blood glucose concentrations also recovered significantly from 58.3 mg/dL during starvation to 166 mg/dL after 72 h of refeeding (Fig. 8c). Glucose levels increased across all regions, with significant restoration observed in the hippocampus and cerebellum (Fig. 8d). Additionally, both the ammonia and urea levels markedly decreased within 24 h of refeeding (Fig. 8e, f).

Consistent with our findings in vitro, SRF-, MRTF-A-, and AD-related markers in the mouse brain were significantly and time-dependently downregulated after refeeding (Fig. 8g–i, Supplementary Fig. 14). The expression of SRF and MRTF-A declined across all brain regions and was substantially reduced after 72 h of refeeding (Fig. 8g, h, Supplementary Fig. 14a, b). The expression of APOE also significantly decreased in the cerebral cortex and hippocampus after refeeding (Fig. 8g, h, Supplementary Fig. 14a, b).

Importantly, AD- and neuroinflammation-associated pathologies, including Aß accumulation, increased pTau/Tau ratios, and iNOS expression, were significantly reversed in all brain regions at 72 h of refeeding. Reactive astrocytic markers, including GFAP and vimentin, were significantly downregulated (Fig. 8i, Supplementary Fig. 14a, b). In contrast, microglial activation markers, including Iba1 and CD68, exhibited only a modest, non-significant reduction in the cerebral cortex (Fig. 8i, Supplementary Fig. 14c, d). However, the decreased expression of iNOS, which is related to neuroinflammation and microglial activation, indicated reduced microglial activation.^29^ The significantly downregulated expression of MMP14, CTSD, LAT1, and ASCT2 in the mouse brain tissue following refeeding, indicating reduced ECM degradation and metabolic reprogramming (Supplementary Fig. 14e, f).

These findings confirmed that hypoglycemia-induced AD-like features were gradually reversed in the mouse brain after restoring glucose. Similar to our 3D model in vitro, restoring glucose at 24 h temporally lagged in fully reversing SRF/MRTF-A expression, which was potentially attributable to transient metabolic memory before neuronal homeostasis was completely restored. We concluded that neurons rely on SRF/MRTF-A-mediated metabolic reprogramming to survive hypoglycemic stress, a response that promotes urea production and neurodegeneration. Excess urea and its derivatives exacerbated astrocyte reactivity and microglial activation, establishing a cascade of pathological events (Fig. 8j).

## Discussion

Severe hypoglycemia, which can result from prolonged fasting or intensive diabetic therapy, leads to neuroglycopenia (glucose deficiency in the brain) and widespread energy deprivation in the brain.^38,39^ The brain ECM plays critical roles in neuronal survival, nutrient uptake, and metabolic regulation.^40–42^ Therefore, the ECM-recapitulating system is essential for accurately modeling brain metabolic disorders in vitro.^43^ Here, we investigated the effects of acute glucose deprivation on the brain in hypoglycemic models in vitro and in vivo. Our results showed that hypoglycemia induces an AD-like pathology, namely amyloid accumulation, tau hyperphosphorylation, and activation of astrocytes and microglia. Using our 3D hypoglycemic model in vitro, we clarified the cell-autonomous responses and molecular mechanisms of neurons, astrocytes, and microglia per se, independent of whole-body systemic factors in vivo. This approach facilitated the elucidation of the molecular pathways involved in neuronal metabolic reprogramming, glial activation, and ECM-related adaptations during glucose deprivation. These results provided insights into how hypoglycemia contributes to neurodegenerative processes.

During starvation, the body initiates an adaptive defense mechanism to counteract hypoglycemia and maintain cellular energy homeostasis. We first discovered the critical role of SRF and its coactivator MRTF-A in an adaptive response to glucose deprivation. The activation of SRF/MRTF-A enables neurons to exploit the surrounding ECM and metabolize amino acids, such as glutamate and glutamine, as alternative fuel sources,^44^ for their survival. Under hypoglycemic stress, neurons enzymatically degrade ECM components, which are subsequently internalized *via* amino acid transporters and processed through intracellular metabolic pathways. Although this mechanism supports neuronal viability, this adaptive process paradoxically leads to neurodegenerative features, such as amyloidogenesis and tau hyperphosphorylation. The brain contains numerous proteases, including MMP14 and cathepsins,^45,46^ that degrade neurotoxic fragments such as Aß, and regulate AD-related pathophysiology. Our results suggest that SRF functions as a transcription factor, orchestrating the expression of ECM-degrading enzymes, amino acid transporters, and neurodegenerative markers, thus linking metabolic adaptation to pathological outcomes. These results reveal a dual role of SRF/MRTF-A in neuronal survival and AD-like neurodegeneration, offering new insights into the molecular interplay between metabolic stress and the neurodegenerative process.

The activation of SRF/MRTF-A represented a neuron-specific adaptive response to hypoglycemia in both in vitro and in vivo models. Our findings indicate that neurons leverage surrounding ECM components, particularly collagen type IV, in an SRF/MRTF-A-dependent manner to sustain survival under glucose deprivation. In contrast, astrocytes maintain viability during hypoglycemia despite low SRF and MRTF-A levels, suggesting an alternative survival mechanism, whereas microglia lacking SRF/MRTF-A activity fail to utilize ECM resources, which reduces their survival under glucose-depleted conditions. These findings highlight a cell type-specific divergence in adaptive metabolic strategies within the brain. Although our study primarily focused on collagen components, the potential contributions of other ECM molecules to cellular energy metabolism remain largely unexplored. Further mechanistic investigations are warranted to determine how other ECM components support neuronal and glial survival during energy stress. This might reveal novel targets to enhance brain resilience under hypoglycemic or metabolic stress conditions.

As part of metabolic reprogramming under glucose deprivation, enhanced amino acid catabolism produces ammonia, which is subsequently detoxified into urea *via* the urea cycle.^36^ Although ammonia-induced neurotoxicity is established, the pathological impact of urea accumulation in the brain has not been studied in detail. Elevated urea levels are linked to several neurodegenerative diseases,^34,35^ potentially disrupting neuronal integrity and exacerbating cognitive impairment. Herein, we found that hypoglycemic stress significantly increased urea and its metabolic derivatives ornithine and putrescine, which in turn triggered astrocyte reactivity and microglial activation, accompanied by elevated expression of inflammatory markers, including iNOS and NFκB.^37,47^ These findings revealed that urea and its downstream metabolites actively contribute to neuroinflammatory and neurodegenerative cascades under hypoglycemia. Therefore, modulation of the urea cycle and its associated pathways might be a promising therapeutic approach to mitigate hypoglycemia-induced neurotoxicity and preserve brain homeostasis.

We previously demonstrated that glucose normalization ameliorates neurodegenerative features in a neurovasculature model of hyperglycemia.^21^ Here, we found that glucose restoration after prolonged deprivation reverses neurodegenerative signatures and attenuates urea production. Clinical evidence indicates that acute malnutrition is associated with reduced gray and white matter in the human brain, which is largely reversible with nutritional rehabilitation.^48–50^ Consistent with this, we showed the progressive recovery of neuronal integrity and molecular homeostasis after glucose restoration, accompanied by a gradual decline in SRF/MRTF-A transcriptional activity. However, this gradual deactivation suggests a transient response in which prior metabolic stress continues to influence cellular responses even after glucose levels return to normal. Although our findings indicate that hypoglycemia-induced neurodegeneration can be reversed, the molecular dynamics of repeated glucose fluctuations and long-term oscillations remain poorly understood. Future investigations should determine whether chronic or cyclic hypoglycemic events result in irreversible neuronal damage, thus potentially contributing to the pathogenesis of progressive neurodegenerative disorders.

Inhibition of SRF/MRTF-A markedly attenuated metabolic reprogramming and neurodegenerative features under hypoglycemic conditions; however, it concurrently caused a pronounced reduction in neuronal survival. These findings suggest that targeting SRF/MRTF-A alone is insufficient, highlighting the need for combined strategies to preserve neuronal viability and mitigate neurodegeneration during glucose deprivation. To elucidate the underlying mechanisms, further studies of SRF/MRTF-A-depleted cells and/or mouse models in vivo under hypoglycemia are warranted. Moreover, future investigations should aim to define the critical threshold at which stressed or neurodegenerative neurons progress to neuronal death, providing insight into potential therapeutic windows for intervention.

In summary, we showed that hypoglycemia activates SRF/MRTF-A-mediated neurodegenerative pathways in in vitro and in vivo models. The neuron-specific activation of SRF/MRTF-A serves as a key adaptive mechanism, promoting metabolic reprogramming through ECM utilization to survive under glucose deprivation. However, this adaptive response paradoxically leads to urea accumulation and exacerbated AD-like neurodegenerative features. Importantly, these pathological changes can be reversed by restoring glucose levels, which coincides with the suppression of SRF/MRTF-A transcriptional activity. Collectively, our results emphasize the critical importance of glucose homeostasis in maintaining neuronal integrity, highlighting SRF/MRTF-A as a central molecular hub connecting metabolic adaptation to neurodegenerative processes. These findings provide a mechanistic foundation for the development of targeted therapeutic strategies aimed at preventing hypoglycemia-induced brain injury and preserving neuronal resilience in metabolic disorders with diabetes.

## Materials and methods

### Cell culture

#### Neuronal cell culture and differentiation

ReN VM human neural progenitor (ReN) cells (SCC008; Merck KGaA, Darmstadt, Germany) were cultured in T75 flasks coated in Matrigel basement membrane matrix (356234; Corning Inc., Corning, NY, USA) in Dulbecco’s modified Eagle’s medium DMEM and Ham’s F-12 (F12) proliferation medium (Gibco 11320-033; Thermo Fisher Scientific Inc., Waltham, MA, USA) supplemented with 1% penicillin‒streptomycin-amphotericin B mix (17-745E; Lonza Group AG, Basel, Switzerland), 2% B27 (Gibco 17504-044; Thermo Fisher Scientific Inc.), 2 µg/mL of heparin (H3149, Sigma-Aldrich Corp., St. Louis, MO, USA), 20 ng/mL of epidermal growth factor (EGF, GF144; Merck KGaA), and 20 ng/mL of fibroblast growth factor (FGF, GF003, Merck). ReN cells were subcultured in Accutase Cell Detachment Solution (SCR005; Merck KGaA).

#### Astrocyte culture

Primary human astrocytes (HAs, #1800; ScienCell Research Laboratories, San Diego, CA, USA) were cultured on T75 flasks coated in poly-l-lysine (2 μg/cm^2^, P4832, Sigma-Aldrich Corp.) in astrocyte medium (#1801), supplemented with 2% fetal bovine serum (FBS; #0010), 1% astrocyte growth supplement (#1852) and 1% penicillin/streptomycin (P/S; #0503; all from ScienCell Research Laboratories) We sub-cultured HAs in 0.05% trypsin/EDTA (T/E; Gibco 25200056; Thermo Fisher Scientific Inc.).

#### Microglia culture

The immortalized human microglia-SV40 cell line (HM) Applied Biological Material Inc. (T0251, Richmond, BC, Canada) was cultured in DMEM/F12 (Gibco 11320-033; Thermo Fisher Scientific Inc.), supplemented with 10% FBS (Gibco 26140079) and 1% P/S (Gibco 15140122), and then subcultured in 0.25% T/E (Gibco 25200056; Thermo Fisher Scientific Inc.). The medium was changed every 2–3 days.

### Three-dimensional cell culture

Matrigel and collagen type I (rat tail, BD Science, United States) were used as biochemical components for ReN cell differentiation^20,21,51^ and to study metabolic alterations under starvation conditions. Collagen type 1 was neutralized with 10× DMEM (D2429) and 1N sodium hydroxide (NaOH; S2770; both from Sigma-Aldrich Corp.) to generate a collagen matrix of 6 mg/mL, then Matrigel was mixed at a proper ratio (Col/Mat; 5:4 ratio). The ReN cells, HA, and HM cells were mixed with a Col/Mat solution at a final cell density of 5 × 10^6^, 2 × 10^6^, and 5 × 10^4^ cells/mL, respectively. The cell-laden collagen/Matrigel solution was incubated at 37 °C for 1 h to complete gelation, then fresh medium was added to the scaffold. The ReN cells were differentiated in proliferation medium without EGF or FGF for 10–14 days at 37 °C in a 5% CO_2_.

### Conditions for glucose starvation and restoring

We established glucose starvation in SILAC Advanced DMEM/F12 Flex medium without glucose (Gibco A2494301), supplemented with 365 mg/L of L-glutamine (25030149), 91.25 mg/L of L-lysine (L8662; all from Thermo Fisher Scientific Inc.), and 147 mg/L of L-arginine (A6969; Sigma-Aldrich Corp.). D-glucose (30 mM, G7021, Sigma-Aldrich Corp.) was added to the SILAC Advanced DMEM/F12 Flex medium for the steady-state control during the experimental period. After ReN differentiation and HA and HM culturing, the medium was changed to SILAC Advanced DMEM/F12 Flex medium with or without D-glucose for 24–48 h. For glucose refeeding experiments, the glucose-depleted medium was changed to a D-glucose-supplemented medium at the indicated time.

### Animal study

The Institutional Animal Care and Use Committee at the Korea Institute of Radiological and Medical Sciences (KIRAMS) approved the protocols that included animals (IACUC No: kirams2024-0136). Six-week-old male C57BL/6 N mice that are widely used in metabolic research^52^ (Doo Yeol Biotech, Yongin, Korea) were housed at the animal research facility of KIRAMS and maintained at 23 °C ± 2 °C, 50% ± 10% humidity, and 12-h light/dark cycle). All mice were acclimated for 5 days before experimentation and had access to standard rodent feed ad libitum and autoclaved water. The C57BL/6 N mice (24 mice in total) were randomly assigned to the following three groups (n = 8 each): (i) ad libitum diet (AL; control), (ii) 24 h starvation, and (iii) 72 h starvation. The starved groups had access only to water. For the refeeding study, 36 mice were randomly assigned to four groups as follows: (i) 72 h of starvation, (ii) 24 h of refeeding, (iii) 72 h of refeeding after 72 h of starvation, and (iv) AL diet. Mice in poor condition, such as those with injuries, were excluded from further studies. All mice were weighed at each experimental time point. Blood glucose concentrations were measured before and after the experiments *via* tail vein prick using an Accu-Check instant blood glucose meter (Roche Diabetes Care, Inc., Basel, Switzerland). Thereafter, 3–4 mice were randomly selected from each group and sacrificed for brain tissue analysis. Whole brains were divided longitudinally into the right hemisphere that was stained with immunofluorescence, and the left hemisphere that was divided into the cerebral cortex, hippocampus, and cerebellum for western blotting and analysis of glucose, ammonia, and urea levels.

### Visualization of degraded DQ-collagen

Fluorescein-conjugated DQ^TM^ Collagen, type I from bovine skin (Invitrogen D12060) and type IV from the human placenta (Invitrogen D12052; Thermo Fisher Scientific Inc.) were used to visualize degraded collagen in media without and with glucose. We incubated HN, HA, and HM cells in 2D plates containing culture medium with 10 µg/mL of DQ-collagen, types I and IV for 24 to 48 h. In the 3D culture, DQ-collagen was mixed with a collagen/Matrigel matrix at a ratio of 1:200. Fluorescence images were acquired using a Zeiss LSM880 confocal laser-scanning microscope (Carl Zeiss AG, Oberkochen, Germany).

### Antibodies and chemicals

Anti-p-Tau Ser202Thr205 (Invitrogen AT8, MN1020) and anti-ß-amyloid (Invitrogen 71-5800; both from Thermo Fisher Scientific Inc.), anti-ß-amyloid (8243; Cell Signaling Technology, Danvers, MA, USA) for mouse analysis), anti-SRF (#5147, Cell Signaling Technology), anti-MRTF-A (sc-390324, Santa Cruz Biotechnology Inc., Santa Cruz, CA, USA), anti-Iba1 (Invitrogen PA5-27436), anti-collagen IV (ab6586; Abcam, Cambridge, UK), and anti-GFAP (G3893, Sigma-Aldrich Corp.) antibodies were used for immunofluorescence staining and western blotting, respectively. Anti-MAP2 (ab5392; Abcam) and anti-Neurofilament light (NF-L; CH22105, Neuromics, Edina, MN, USA) were used for immunofluorescence staining. Anti-Tau (sc-166060), anti-cathepsin D (sc-377299), anti-CD98 (sc-59145), all from Santa Cruz Biotechnology Inc), anti-MMP14 MAB918 (R&D Systems, Minneapolis, MN, USA), anti-SLC1A5 (MBS7045915, MyBioSource Inc., San Diego, CA, USA), anti-SLC7A5 (Invitrogen PA5-50485; Thermo Fisher Scientific Inc.), anti-CD68 (76437, Cell Signaling Technology), anti-CD40 (ab13545), anti-CD11b (ab133357), Vimentin (ab92547; all from Abcam), anti-APOE (Invitrogen 701241), anti-iNOS (Invitrogen PA1-036) and HRP-linked anti-mouse IgM (Invitrogen 31440; all from Thermo Fisher Scientific Inc.), anti- anti-NFκB (sc-8008), anti-Collagen I anti-ß-Actin (sc-47778, Santa Cruz), anti-ß-tubulin (sc-5274), and anti-Lamin A/C (sc-7292, all from Santa Cruz) Biotechnology Inc.), (NB600-408, Novus), horseradish peroxide (HRP)-linked anti-rabbit IgG (7074, Cell Signaling), and HRP-linked anti-mouse IgG (7076, Cell Signaling Technology) antibodies were used for western blotting. Phalloidin tetramethyl rhodamine B isothiocyanate (P1951) and 4,6-diamidino-2-phenylindole (DAPI, D9564; both from Sigma-Aldrich Corp.), Alexa Fluor 488 goat anti-mouse IgG (Invitrogen A-11001) and Alexa Fluor 594 goat anti-rabbit IgG (Invitrogen A-11012), and Alexa Fluor 647 goat anti-chicken IgY (Invitrogen A-21449, Thermo Fisher Scientific Inc.) were used for immunofluorescence staining.

NSC405020 (S8072) and pepstatin A (S7381; both from SelleckChem, Houston, TX, USA), SelleckChem) were used to inhibit ECM proteolysis. CCG-203971 (SML1422; Sigma-Aldrich Corp.) was used to inhibit SRF/MRTF-A expression. Chemicals were diluted in neuronal differentiation medium to the described concentrations and incubated for 48 h. Urea (U5378), L-ornithine monohydrochloride (O6503), and putrescine dihydrochloride (P5780; all from Sigma-Aldrich Corp), and amyloid-ß peptides 1-40 and 1-42 (21617 and 20574, respectively; Cayman Chemical Company Inc., Ann Arbor; MI, USA) were diluted in culture media containing glucose (5.5 mM, 100 mg/dL) at the described concentrations and incubated for 48 h.

### Cell viability assay

Cell viability was analyzed using WST-8 Cell Viability Assay Kits (QM1000; Biomax Co. Ltd., Guri Si, South Korea). HN, HA, and HM cells cultured on Matrigel-coated 48-well plates and in a 3D matrix were incubated with WST-8 solution for 30 min and 1 h, respectively, as described by the manufacturer. Viability was assessed by measuring absorbance at 450 nm using a Synergy HTX Multi-Mode Microplate Reader (BioTek Instruments, Winooski, VT, USA).

We used LIVE/DEAD Viability/Cytotoxicity Kits (Invitrogen L3224; Thermo Fisher Scientific Inc.), which contain Calcein-AM and ethidium homodimer-1 (EthD-1) to stain live and dead cells, respectively. The viability of cells cultured without glucose was determined by incubating 3D models with staining solution for 1 h. Fluorescence images of live and dead cells in the 3D culture model were captured using an LSM880 confocal laser-scanning microscope (Carl Zeiss), and cell viability was quantified by counting live and dead cells.

### Immunofluorescence staining

Cells on 2D plates and in a 3D matrix were fixed with 4% paraformaldehyde (P2031, Biosesang, Yongin Si, South Korea), permeabilized with 0.2% Triton X-100 (Sigma-Aldrich Corp.), and non-specific protein binding was blocked with 2 wt% bovine serum albumin (BSA, A0100-010; GenDEPOT, Baker, TX, USA). The samples were incubated with primary, followed by secondary antibodies (1:1000; Invitrogen, Thermo Fisher Scientific Inc.) overnight at 4 °C. Fluorescence images were acquired using the LSM880 confocal laser-scanning microscope (Carl Zeiss AG.).

### Image-based quantification and calculation

#### Normalization of western blot band intensity

The band intensity was measured and quantified using ImageJ2 software (v.2.14.0) and normalized to that of the loading control. The normalized values were calculated as fold changes based on the control group for better comparison. Statistical analyses were performed using more than three independent biological replicates.

#### Normalization of fluorescence intensity

Fluorescent images of the tissue section and in vitro model were analyzed using ImageJ software to quantify the fluorescence intensity of the target proteins. Nuclei were stained with DAPI, and the number of nuclei was determined through particle analysis. The fluorescence intensity of the target proteins was measured and normalized to the number of nuclei.

#### Analysis of nuclear localization

ImageJ software was used to quantify the percentage of nuclear SRF in mouse brain cells. We measured the overlapping fluorescence of SRF in the nucleus, and particle analysis was performed to count SRF-positive nuclei. Statistical analyses were performed using more than seven images obtained from three biologically independent mouse brain tissue samples.

#### Calculation of differentiated population

The percentage of differentiated neurons and astrocytes was analyzed using ImageJ software. We calculated the percentage of differentiation by comparing the number of MAP2-positive and GFAP-positive cells in neurons and astrocytes with the total number of nuclei (DAPI).

### Protein expression

Total proteins from HN, HA, and HM cells grown on 2D plates were extracted using radioimmunoprecipitation assay (RIPA) buffer (R4200, GenDEPOT) supplemented with Xpert Duo Inhibitor Cocktail Solution (P3300, GenDEPOT). To analyze intracellular protein expression in the 3D matrix, cells were recovered by removing the collagen/Matrigel matrix with collagenase type 1 (Gibco 17018029; Thermo Fisher Scientific Inc.) and cell recovery solution (354253, Corning Inc.). Recovered cells and brain tissues were disrupted using RIPA cell lysis buffer supplemented with protease and phosphatase inhibitors. Nuclear and cytoplasmic fractionation was analyzed using NE-PER Nuclear and Cytoplasmic Extraction Reagents (7883; Thermo Fisher Scientific Inc.) as described by the manufacturer. The concentrations of the extracted proteins were measured using Bradford assays. The proteins (10 µg) were resolved by SDS-PAGE and transferred to NitroPure nitrocellulose membranes (LC7033-300, GenDEPOT) and blotted with primary and HRP-conjugated secondary antibodies. Protein expression was detected using West-Q Pico Dura ECL solution (W3653; GenDEPOT) and chemiluminescent images were captured using a ChemiDoc MP Imaging System (12003154, Bio-Rad Laboratories Inc., Hercules, CA, USA). Alexa Fluor 488 mode was applied for imaging of DQ^TM^ Collagen in cell culture media.

Amyloid β40 and β42 in mouse cerebral cortices were analyzed using Aβ40 (Invitrogen KMB3481) and Aβ42 (Invitrogen KMB3441) ELISA assay kits (both from Thermo Fisher Scientific Inc.) as described by the manufacturer.

### Analysis of mouse brain sections

The half brain was fixed in 4% paraformaldehyde (P2031; Biosesang), embedded in paraffin, and sliced into 4-µm-thick coronal sections using a rotary microtome (HM325; Thermo Fisher Scientific Inc.). The sections were deparaffinized and rehydrated then heat-induced antigen retrieval proceeded in citrate buffer (H-3300-250; Vector Laboratories, Inc., Newark, CA, USA). Non-specific proteins in the sections were blocked with 5% BSA and incubated overnight at 4 °C with primary antibodies diluted as follows: ß-Amyloid (1:1000), pTau (1:100), MAP2 (1:5000), NF-L (1:2000), Vimentin (1:1000), GFAP (1:1000), Cathepsin D (1:100), Collagen IV (1:1000), SRF (1:1000), MRTF-A (1:100), and Iba1 (1:500). The sections were then incubated with Alexa Fluor 488-, 594-, or 647-conjugated secondary antibodies (1:1000), and the nuclei were counterstained with DAPI (1:2000). The stained brain sections were mounted with Fluoromount-G (0100-01, SouthernBiotech, Birmingham, AL, USA) and coverslipped (Paul Marienfeld GmbH & Co. KG., Lauda-Königshofen, Germany). Fluorescence images of cerebral cortex and hippocampal DG regions were acquired using a fluorescence microscope (Carl Zeiss AG.).

### Analysis of intracellular glucose, urea, and ammonia

Levels of intracellular glucose, urea, and ammonia were determined using a PicoSens Glucose (BM-GLO-100, Biomax Co. Ltd.), an Ammonia (K-AMIAR; Megazyme, Wicklow, Ireland), and a QuantiChrom^TM^ Urea Assay Kit DIUR-100 (BioAssay Systems, Hayward, CA, USA), respectively, in the hypoglycemic 3D model and mouse brain tissues. We homogenized ~4 × 10^5^ cells embedded in 3D hydrogels in 50 µL of triple-distilled water and lysates (10 mg/mL) from mouse brain regions were prepared.

#### Calculation of glucose concentrations

Glucose concentrations in extracts were determined by measuring absorbance at 570 nm using a Synergy HTX Multi-Mode Microplate Reader (BioTek Instruments). The intracellular glucose concentration was calculated by comparison with a standard curve.

#### Calculation of ammonia concentrations

Ammonia concentrations in extracts were determined by measuring absorbance at 340 nm using a microplate reader. Intracellular ammonia is presented as relative values compared with control.

#### Calculation of urea concentrations

These were determined by measuring absorbance (A) at 520 nm using a microplate reader as follows:

1 $$\mathrm{Urea\; concentration}\,(\mathrm{mg}/\mathrm{dL})=({{\rm{A}}}_{\mathrm{Sample}}-{{\rm{A}}}_{\mathrm{Blank}}/{{\rm{A}}}_{\mathrm{Standard}}-{{\rm{A}}}_{\mathrm{Blank}})\times \mathrm{standard}\,50$$

### RNA isolation and sequencing

Total RNA from 3D-differentiated ReN cells under hypoglycemic and steady-fed conditions for 24 and 48 h was isolated using QIAzol lysis reagent (79306; Qiagen GmbH, Hilden, Germany). The RNA preparation and sequencing methods for RNA-seq analysis are detailed in our previous report.^20^ Briefly, total RNA quality and quantity were assessed using an Agilent 2100 Bioanalyzer (Agilent Technologies, Santa Clara, CA, USA) and a NanoDrop-2000 spectrophotometer (Thermo Fisher Scientific Inc.). We isolated mRNA using Poly(A) RNA Selection Kits (Lexogen, Vienna, Austria) and prepared RNA libraries using NEBNext Ultra II Directional RNA-Seq Kits (New England BioLabs, Ipswich, MA, USA). Complementary cDNA was synthesized, then synthesized, fragmented, and indexed, libraries were amplified by PCR and evaluated using TapeStation HS D1000 Screen Tape (Agilent Technologies) and StepOne Real-Time PCR System (Thermo Fisher Scientific Inc.). Amplicons of interest were sequenced using a NovaSeq 6000 (Illumina, San Diego, CA, USA) platform with paired-end 100 bp reads. Raw reads were quality-checked using FastQC, and low-quality or adapter sequences were removed with FASTX_Trimmer and BBMap. Trimmed reads were aligned to the reference genome using TopHat software. Gene expression was quantified as FPKM values using Cufflinks, with normalization by EdgeR in R.

### Bioinformatics analysis

We analyzed Gene Ontology enrichment terms using EnrichR software (http://amp.pharm.mssm.edu/Enrichr/) based on RNA-seq data ranked by fold changes between starvation (0) and HG conditions for 24 and 48 h. *P* values and odds ratios ranked enriched GO terms. A bar plot displays significantly enriched pathways (*p* < 0.05), and a bubble plot shows p values and odds ratios on the two axes. Gene Set Enrichment Analysis (GSEA) was conducted using the Molecular Signatures Database (MSigDB version 7.5.1). The bubble plot of GSEA results displays false discovery rates (FDR) and normalized scores of enriched pathways along the two axes, with bubble size and color representing proportions of overlapping genes.

### Transcriptomic analysis from the GEO database

We used the public GEO database (GSE64226) that includes microarray-based gene expression profiles in the hippocampi of control-fed and calorie-restricted mice (*n* = 5 per group) to identify transcriptomic changes caused by long-term CR by comparing gene expression between the control and CR groups, we performed GO and GSEA analyses, and the enriched pathways were visualized in color-coded bubble plots.

We analyzed transcriptomic changes in brain disorders using microarray-based public GEO data (GSE63060) using blood samples from patients with AD (*n* = 49), mild cognitive impairment (MCI, *n* = 39), and healthy older controls (N; *n* = 67). Transcriptomic of *SLC2A1, SLC2A3, SRF*, and *MKL1/2* mRNA were analyzed, and significance was calculated using one-way analysis of variance with Tukey multiple comparison tests.

### ChIP-Atlas analysis

We visualized protein binding in given genomic regions based on published ChIP-Seq data using the Peak Browser tool in ChIP-Atlas.^31^ The parameter class settings were: antigen, transcription factors and others; cell type, all cell types, and a threshold of 100 for significance (model-based analysis of ChIP-seq [MACS] scores). ChIP peaks near the transcription start sites of selected genes were considered as transcription factor-binding sites. All peak call data recorded in the ChIP-Atlas were graphically displayed using Integrative Genomics Viewer (IGV 2.16.1). Target genes of the SRF transcription factor were obtained from ChIP-seq datasets in the ENCODE transcription factor target datasets (https://maayanlab.cloud/Harmonizome/dataset/ENCODE+Transcription+Factor+Targets). Genes associated with AD were selected from the KEGG pathway, “Alzheimer’s disease (hsa05010)”, and their genomic locations were determined based on the human hg19 reference genome assembly.

### Statistical analysis

Data were analyzed using Prism ver. 10.3 (GraphPad Prism Software, San Diego, CA, USA). Whether a dataset was normally distributed was determined using the Shapiro–Wilk normality test (*p* > 0.05). Paired groups were statistically compared using unpaired two-tailed *t*-tests, and three or more groups were compared using ordinary one-way analyses of variance with Tukey multiple comparison tests. *P* and exact values are represented by asterisks in graphs and are defined in the figure legends. All experiments were performed at least in triplicate, and the number of replicates and statistical methods are also indicated in the figure legends. One RNA sample from each condition was sequenced, and representative values normalized from two measurements were used in the bioinformatics analysis. Box-and-whisker plots show medians (horizontal bars), interquartile ranges (25–75%, box edges), and minimum-to-maximum values (whiskers) for all points. Values in scatter dot plots are presented as means ± standard deviation (SD).

## Supplementary information

Supplementary Material

## Data Availability

All data necessary to understand and evaluate the conclusions of this study are provided in the article and Supplementary Information. Sequencing data are deposited under the NCBI GEO accession number, GSE308305. All other data are available from the corresponding author upon reasonable request.
