# Supplementary material for: Hypoglycemia induces brain metabolic reprogramming and neurodegeneration via serum response factor and myocardin-related transcription factor-A
Source: Signal Transduct Target Ther. 2025 Dec 17;10:412. doi: 10.1038/s41392-025-02527-x (PMC12711915; doi:10.1038/s41392-025-02527-x)
Supplement: Supplementary file 1 — Supplementary Material [file 41392_2025_2527_MOESM1_ESM.docx]

Supplementary Materials for

Hypoglycemia induces brain metabolic reprogramming and neurodegeneration via serum response factor and myocardin-related transcription factor-A

Minjeong Jang*, Hyung Jin Choi, Hae-June Lee, Hong Nam Kim*

Correspondence to: Dr. Minjeong Jang ([jmj.jang@kirams.re.kr](mailto:jmj.jang@kirams.re.kr)), Dr. Hong Nam Kim ([hongnam.kim@kist.re.kr](mailto:hongnam.kim@kist.re.kr))

**This PDF file includes:**

Supplementary Figs. 1 to 14

**
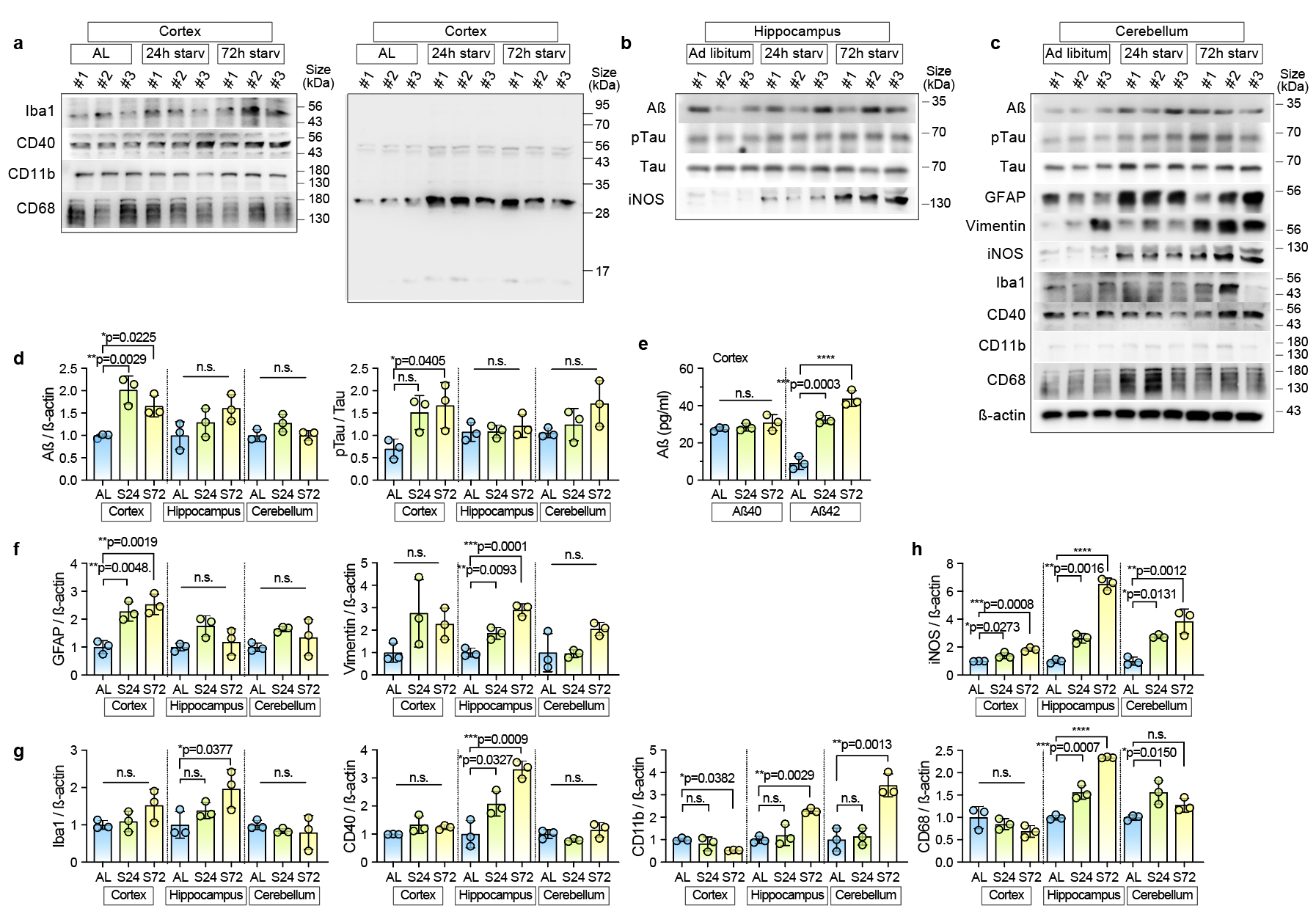
**

**Supplementary Fig. 1. Expression of neurodegenerative markers in mouse brains with glucose depletion.**

**a-c,** Expression of Aß, pTau, Tau, GFAP, vimentin, iNOS, Iba1, CD40, CD11b, and CD68 proteins under *ad libitum* (AL), 24 hours (S24), and 72 hours of starvation (S72) in cerebral cortex (**a**), hippocampus (**b**), and cerebellum (**c**). Uncropped Aß blots with marker size. ß-Actin was used as a loading control, which is identical to that shown in Fig. 1h, i. **d-h,** Comparison of protein expression in cerebral cortex, hippocampus, and cerebellum of mice compared with AL (control). Graphs show expression of Aß and ratio of pTau/Tau (**d**), Aß40 and Aß42 in the cerebral cortex (**e**), GFAP and vimentin (**f**), Iba1, CD40, CD11b, and CD68 proteins (**g**), and neuroinflammation markers, iNOS (**h**) under AL, S24, and S72 conditions. Expression is relative to AL. Scatter dot plots show means ± SD with bars and error bars (n = 3; ****p < 0.0001, ordinary ANOVA followed by Tukey multiple comparison tests, n.s. not significant).

**
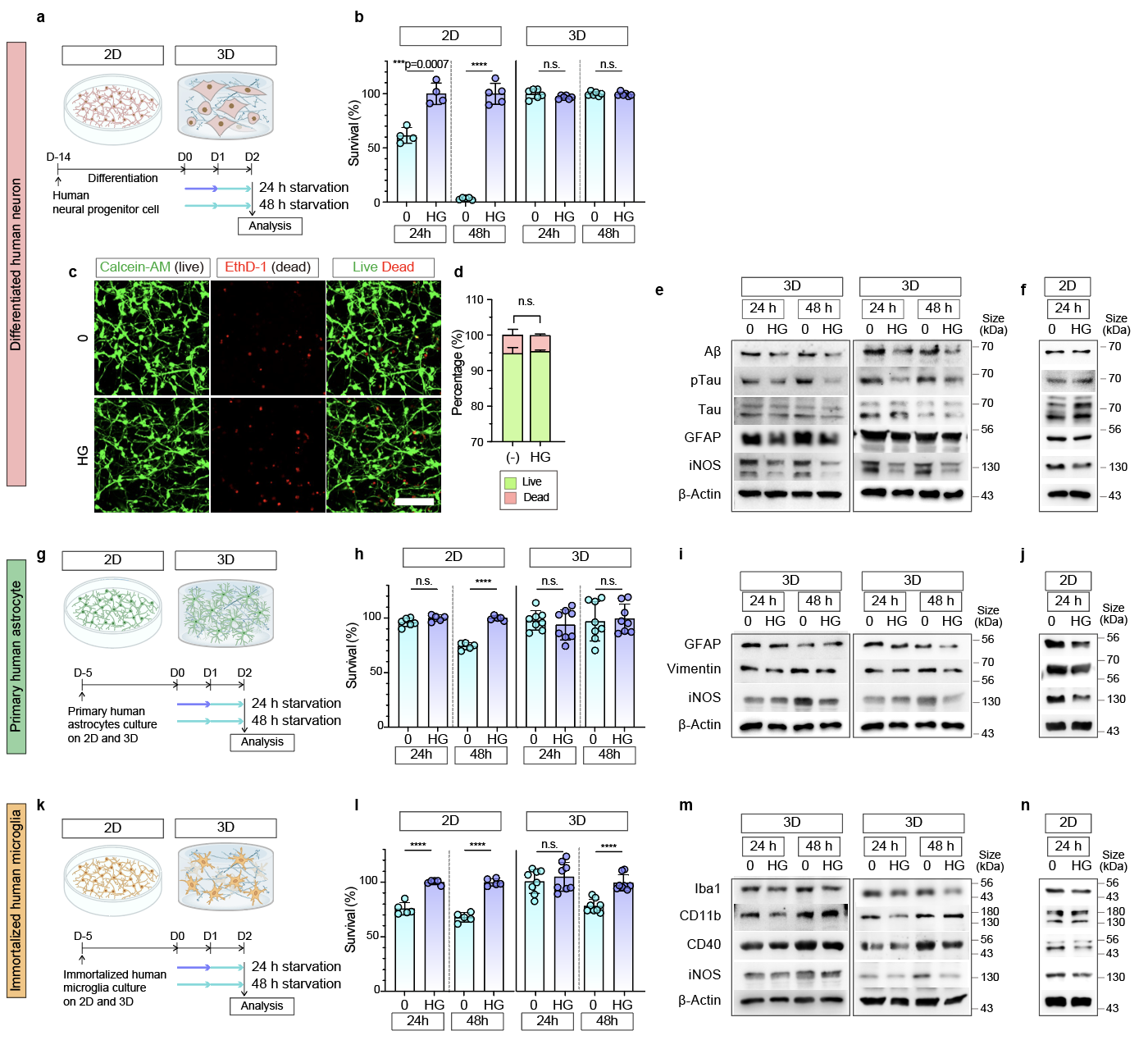
**

**Supplementary Fig. 2. Analysis of cell viability and neurodegenerative features in 2D and 3D hypoglycemic models.**

**a, g, k**, Hypoglycemic models *in vitro* comprise neuronal cells on 2D plates and in 3D ECM hybrid scaffolds of Matrigel and collagen type 1. Human neural progenitor cells (HN, **a**) were differentiated into astrocytes and neurons for 14 days. Primary human astrocytes (HA, **g**) and immortalized human microglia (HM, **k**) were cultured for 5 days on 2D plates and in 3D ECM hybrids. After differentiation and culture, a hypoglycemic environment was created provided by incubating cells in glucose-free media for 24 and 48 hours. **b, h, l,** Survival of cells in hypoglycemic models on the 2D plates and in the 3D ECM hybrid without (0) and with (H) glucose for 24 and 48 h. Survival of HN (**b**), HA (**h**), and HM (**l**) cells was calculated relative to a group given glucose for 24 hours, which was considered 100%. Scatter dot plot shows means ± SD with bars and error bars. Significance was calculated using unpaired t-tests (n=5-8; ****p < 0.0001; n.s.; not significant). **c, d,** Live/dead staining of HN-cultured 3D model after 48 hours of glucose depletion, compared with HG. Calcein AM (green) and Ethidium homodimer (EthD-1, red) were used for live and dead staining, respectively. Scale bar = 100 µm. The number of live and dead cells (**d**) was quantified (n=8, n.s.: not significant). Expression of markers indicating **e,** neurodegeneration and neuroinflammation (Aß, pTau, Tau, GFAP, and iNOS), **i,** reactive astrocytes (GFAP, vimentin, and iNOS), and **m,** microglial activation markers (Iba1, CD11b, CD40, and iNOS) under glucose depletion (0) and HG for 24 and 48 h in HN, HA, and HM cultured in the 3D models. ß-actin was used as a loading control. Western blots of quantified triplicate samples are shown in Fig. 2c, e, g. **f, j, n**, Expression of neurodegenerative (**f**), reactive astrocyte (**j**), and microglial activation (**n**) markers under depleted glucose and HG for 24 hours in HN, HA, and HM cultured on 2D plates. ß-actin was used as a loading control.

**
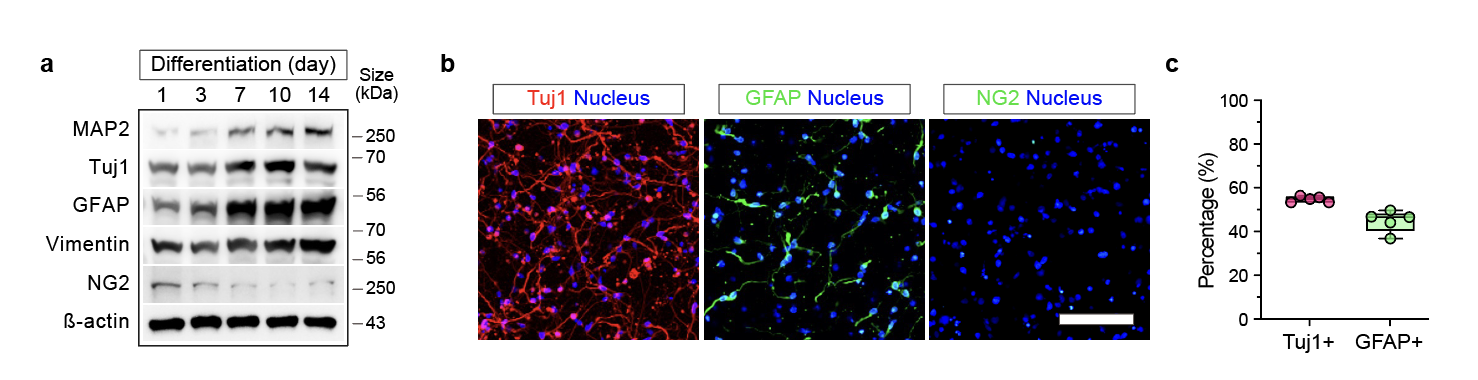
**

**Supplementary Fig. 3. Analysis of ReN cell differentiation in 3D models.**

**a,** Analysis of neuron, astrocyte, and oligodendrocyte markers on differentiation days. ß-actin was used as a loading control. **b,** Immunofluorescence staining of Tuj1 (red), GFAP (green), and NG2 proteoglycan (green) in 3D matrix after 14 days of differentiation. Scale bar = 100 µm. **c,** Rates (%) of differentiation into neurons and astrocytes (n = 5).

ReN cells possess the capacity to differentiate into neurons, astrocytes, and oligodendrocytes.^1^ To characterize the differentiated population within our 3D system, we evaluated the expression levels of differentiation markers at multiple time points (days 1, 4, 7, 10, and 14; **Supplementary Fig. 3a**). Prolonged differentiation resulted in increased expression of neuronal markers (MAP2 and Tuj1) and astrocyte markers (GFAP and Vimentin), plateauing after 10 days. In contrast, oligodendrocyte marker expression (NG2 proteoglycan) declined over time (**Supplementary Fig. 3b)**. Furthermore, oligodendrocyte marker expression was consistently lower than that of neuronal and astrocyte markers (**Supplementary Fig. 3b**). Quantitative analysis of differentiated profiles indicated that approximately 55% of the total cell population were Tuj1-positive neurons and 45% were GFAP-positive astrocytes (**Supplementary Fig. 3c)**.

^1^ Song, Y. *et al.* A dynamic view of the proteomic landscape during differentiation of ReNcell VM cells, an immortalized human neural progenitor line. *Sci Data* **6**, 190016 (2019).

**
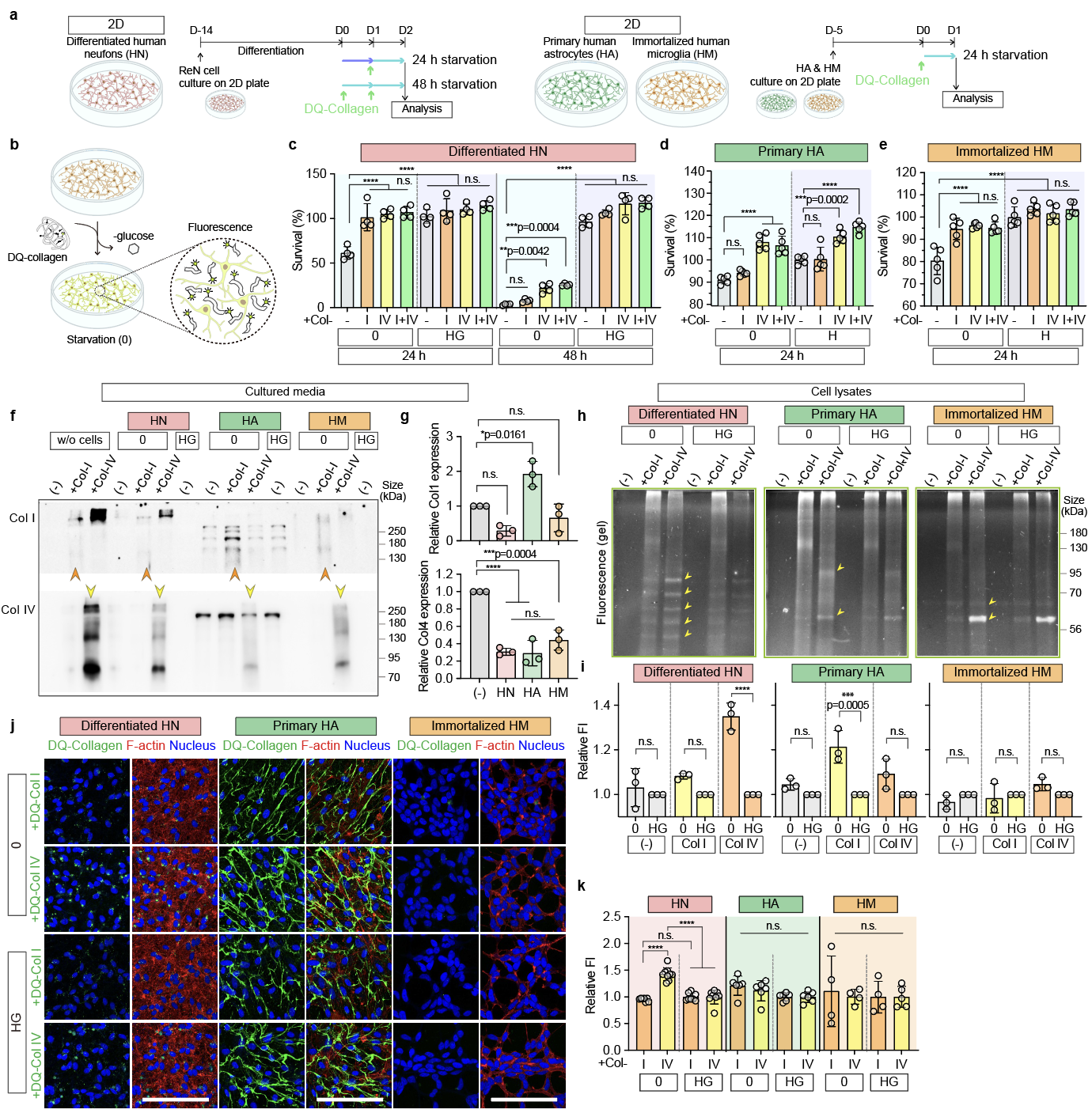
**

**Supplementary Fig. 4. Utilization of ECM as an energy fuel for neuronal survival in neurons with depleted glucose.**

**a,** Schematic illustration of DQ-collagen treatment on 2D hypoglycemic models. After HN differentiation and HA and HM were cultured, cells were incubated in glucose-free media containing DQ-collagen for 24 and 48 h. **b**, DQ-collagen emits fluorescent signals when ECM components are enzymatically degraded. **c-e,** Viability of HN (**c**), HA (**d**), and HM (**e**) cells cultured on 2D models without (0) and with glucose (HG) conditions for 24 and 48 h. Significance was calculated using an unpaired t-test. Data in scatter dot plots are shown as means ± SD with bars and error bars. (HN, n = 4; HA, n = 5; HM, n = 5. ****p < 0.0001; n.s., not significant; one-way ANOVA followed by Tukey multiple comparison tests). **f,** Analysis of DQ-collagen types I and IV in HN, HA, and HM incubated in glucose-free media for 24 h. Changes in the contents of DQ-collagen types I (orange arrow) and IV (yellow arrow) were compared with the medium without cells. **g,** Relative amount of collagen types I and IV in HN, HA, and HM cultured medium containing DQ-collagen types I and IV *versus* medium without cells. We quantified the whole band of collagen I and IV, indicated by orange and yellow arrows in **f**, respectively. Data in scatter dot plots are shown as means ± SD with bars and error bars (n = 3; ****p < 0.0001, ordinary one-way ANOVA, followed by Tukey multiple comparison tests; n.s., not significant). **h,** Fluorescence intensity emitted by degraded DQ-collagen types I and IV in HN, HA, and HM cell lysates without or with glucose. **i,** Fluorescence intensity of degraded DQ-collagen types I and IV in HN, HA, and HM cell lysates relative to HG. Data in scatter dot plots are shown as means ± SD with bars and error bars showing all points (n = 3, ****p < 0.0001, unpaired two-tailed t-tests; n.s., not significant). **j,** Fluorescence images of degraded DQ-collagen types I and IV (green) on 2D cultured HN, HA, and HM without and with glucose (HG) after 24 h. F-actin (red) and nuclei (blue) were stained. Scale bar = 100 µm. **k,** Image-based quantitation of fluorescence intensity of degraded DQ-collagen types I and IV in the 2D-cultured HN, HA, and HM, related to the HG conditions. Data in scatter dot plots are shown as means ± SD with bars and error bars showing all points. Significance was calculated using ordinary one-way ANOVA, followed by Tukey multiple comparison test (n = 7-9 in HN, n=6 in HA, n=6 in HM; ****p < 0.0001; n.s. not significant).


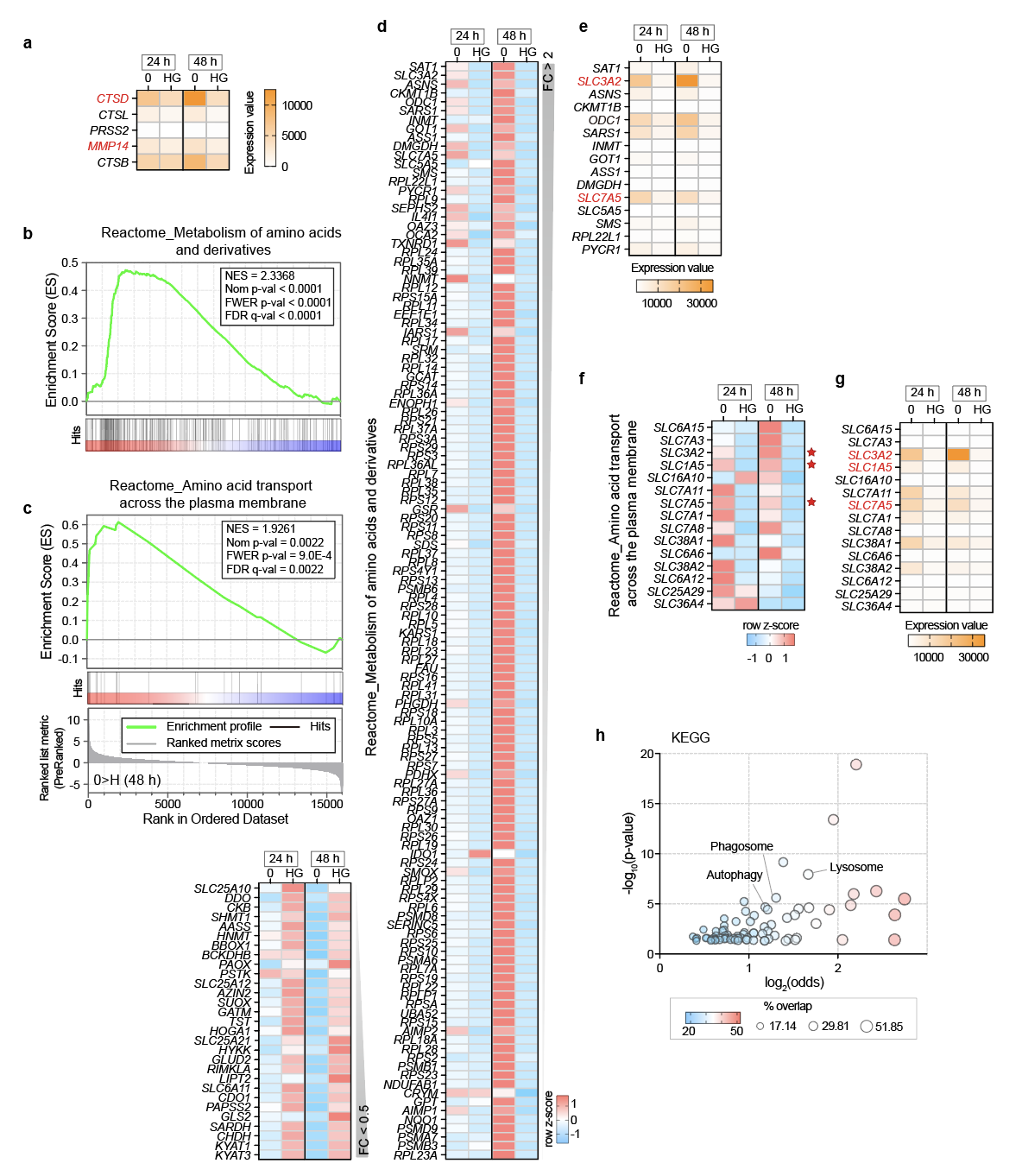


**Supplementary Fig. 5. RNA-seq-based bioinformatics analysis.**

**a**, Heatmap shows expression in the gene set “Collagen degradation” from the Reactome pathway. **b, c,** Gene set enrichment analysis (GSEA) shows significant enrichment of the gene set, “Metabolism of amino acids and derivatives” (**b**) and “Amino acid transport across the plasma membrane” (**c**) from the Reactome pathway in the starvation group for 48 hours compared to the HG group. Red and blue indicate high and low log-ranked values, respectively, for the comparison of starvation (0) to the fed (HG) group. **d-g,** Heatmap based on *z* scores (**d, f**) and expression (**e, g**) shows expression in the gene sets, “Metabolism of amino acids and derivatives” (**d, e**) and “Amino acid transport across the plasma membrane” (**f, g**) from the Reactome pathway. **h**, Gene Ontology (GO) analysis of significantly enriched gene sets from KEGG pathways in the starvation (0) condition compared to the fed (HG) condition. The bubble plot represents significantly enriched gene sets with the odds rate presented as a log_2_ value and the p-value presented as a -log_10_ value.

**
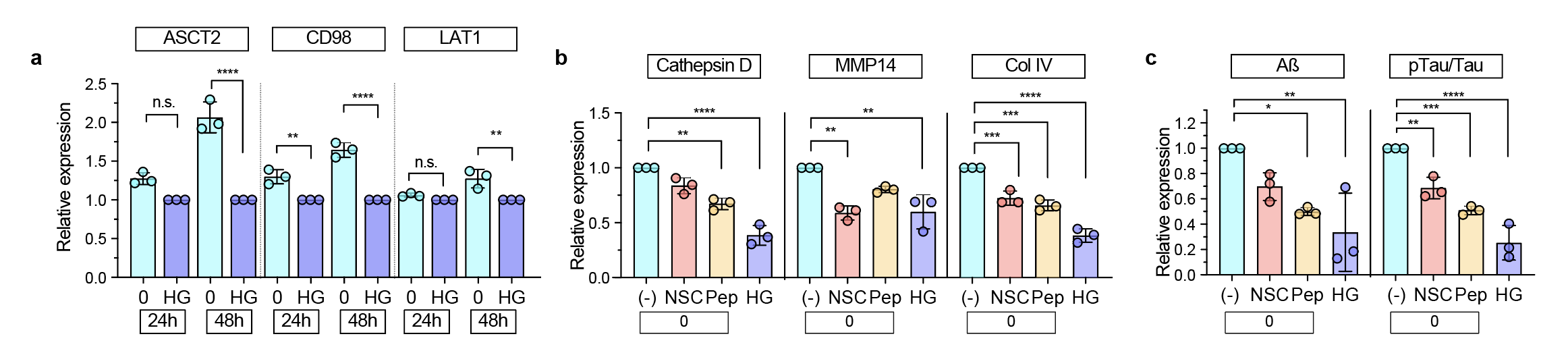
Supplementary Fig. 6. Quantification of protein expression in 3D models.**

Relative expression was calculated from western blot images shown in Fig. 3f (**a**), p (**b**), and u (**c**). Band intensity was normalized by ß-actin and is shown relative to HG groups.


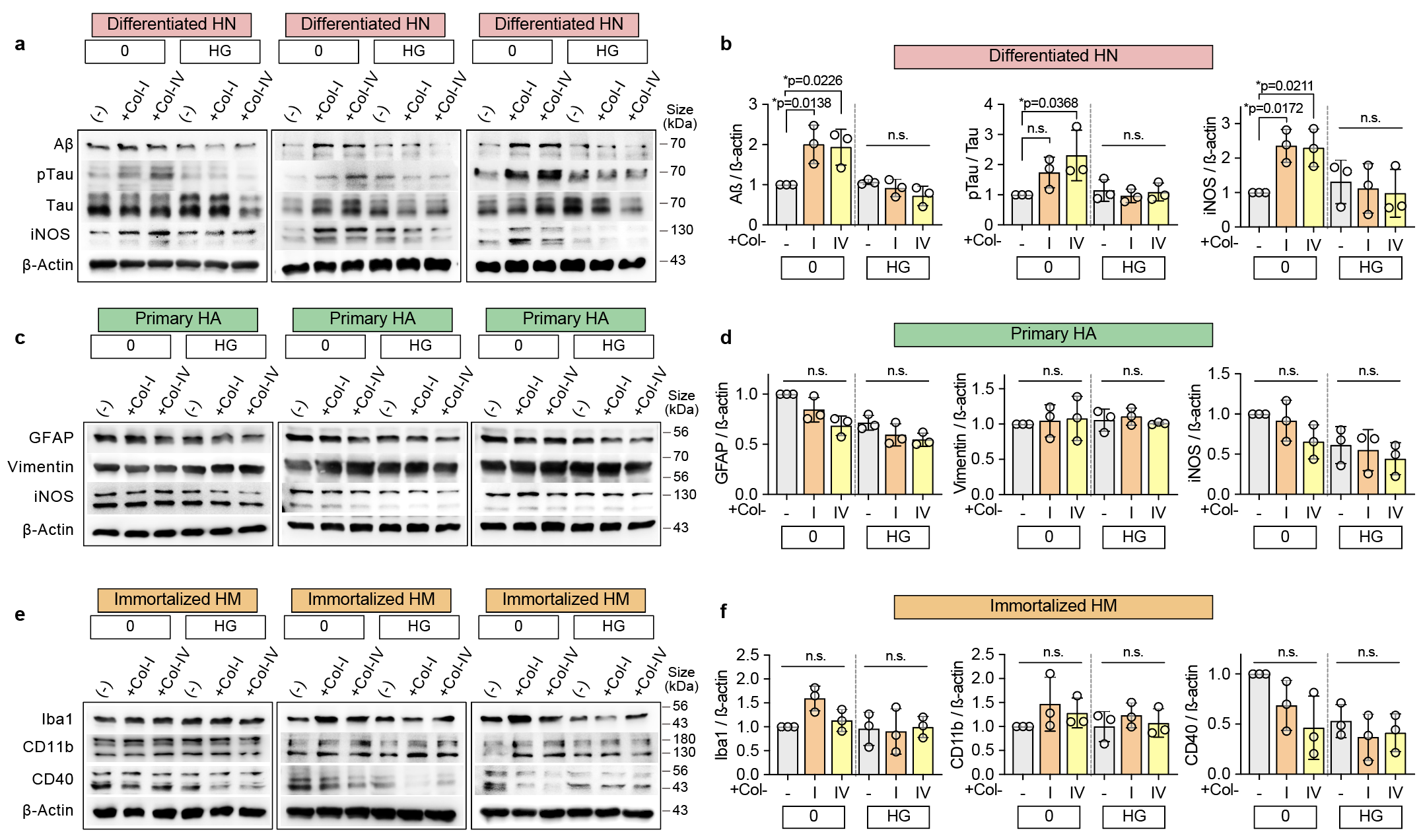


**Supplementary Fig. 7. Effects of ECM on neurodegeneration in 2D hypoglycemic models.**

**a, c, e,** Expression of neurodegenerative markers (**a,** Aß, pTau, Tau, and iNOS), reactive astrocyte markers (**c,** GFAP, vimentin, and iNOS), and microglial activation markers (**e,** Iba1, CD11b, and CD40) proteins by treating DQ-collagen type I and IV under starvation (0) and fed (HG) conditions in differentiated HN- (**a**), primary HA- (**c**), and immortalized HM- (**e**) cultured on the 2D plates. ß-actin was used as a loading control. Western blot analysis was performed in triplicate. **b, d, f,** Quantification of protein expression compared to non-treated (-) DQ-collagen under starved conditions. Graphs represented the relative expression values of neurodegenerative markers (**b,** Aß, ratio of pTau/Tau, and iNOS), reactive astrocyte markers (**d,** GFAP, vimentin, and iNOS), and microglial activation markers (**f,** Iba1, CD11b, and CD40). The scatter dot plots represent the mean ± SD with bars and error bars. Significance was calculated using an ordinary one-way analysis of variance, followed by Tukey’s multiple comparison test (n=3; n.s.; not significant).


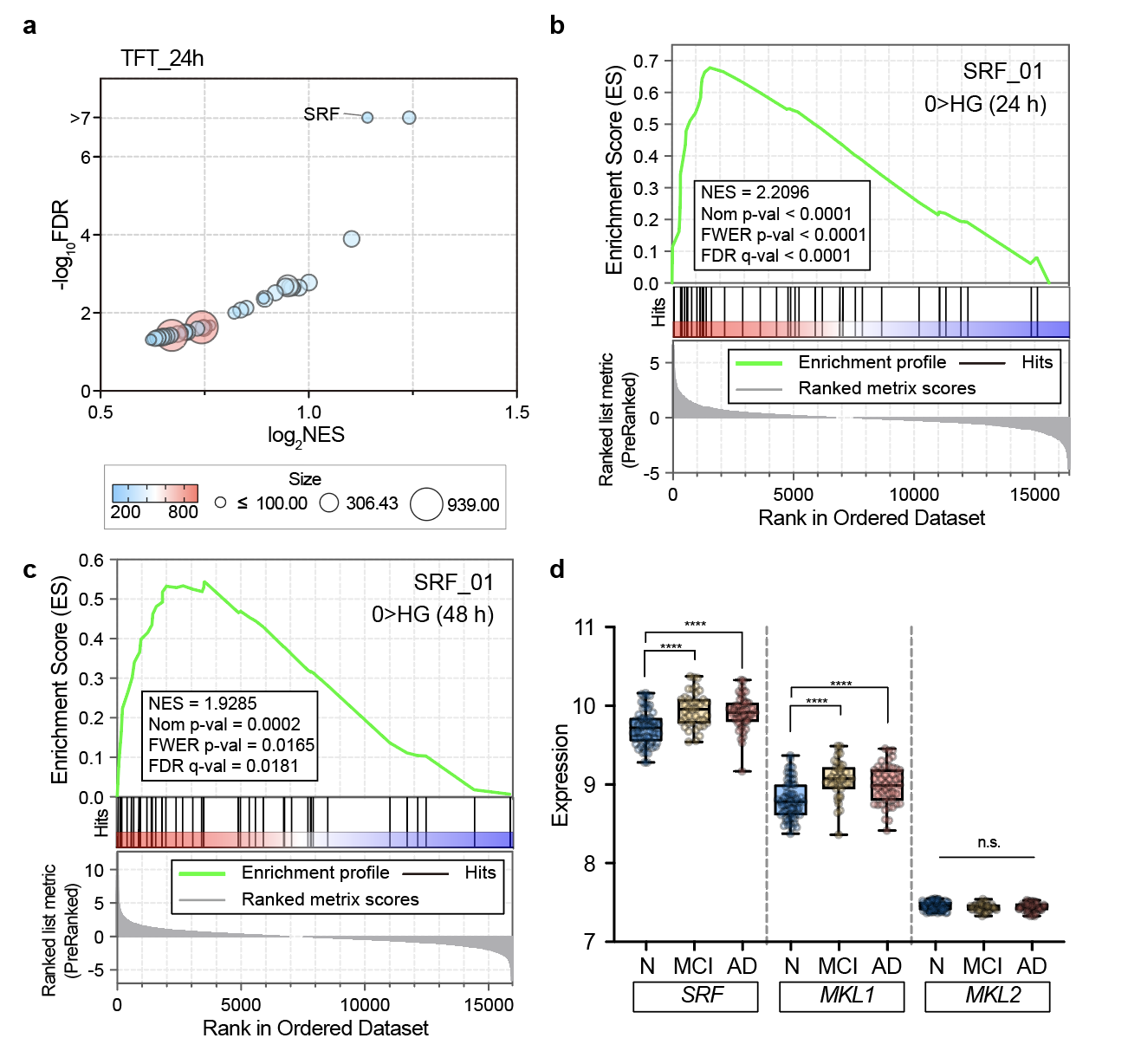


**Supplementary Fig. 8. SRF and MRTF-A are significantly related to hypoglycemia and Alzheimer’s disease.**

**a-c,** Gene set enrichment analysis (GSEA) using TFT_Legacy (legacy transcription factor targets) from the Molecular Signatures Database (MSigDB) showed significant enrichment of SRF in the starvation group for 24 **(a, b)** and 48 hours **(c)** compared to the HG group. The bubble plot **(a)** represents significantly enriched gene sets with a false discovery rate (FDR) < 0.05 and the normalized enrichment score (NES) as a log_2_ value. The red and blue gradations in **b** and **c** indicate high and low log-ranked values, respectively, for the comparison of 0 to the HG group. NES: normalized enrichment score, NOM: nominal, FWER: familywise error rate, FDR: false discovery rate. **d,** The mRNA expression analysis of *SRF* and *MKL1/2* in normal, mild cognitive impairment (MCI), and Alzheimer’s disease (AD) patients in the human GEO database (GSE63063). Box and whisker plots present the median (horizontal bar) and the minimum and maximum values with all data points. Significance was determined by one-way ANOVA with Tukey’s multiple comparisons tests (n = 67 in the normal group, n = 39 in the MCI group, n = 49 in the AD group; *****p*<0.0001, n.s.: not significant).


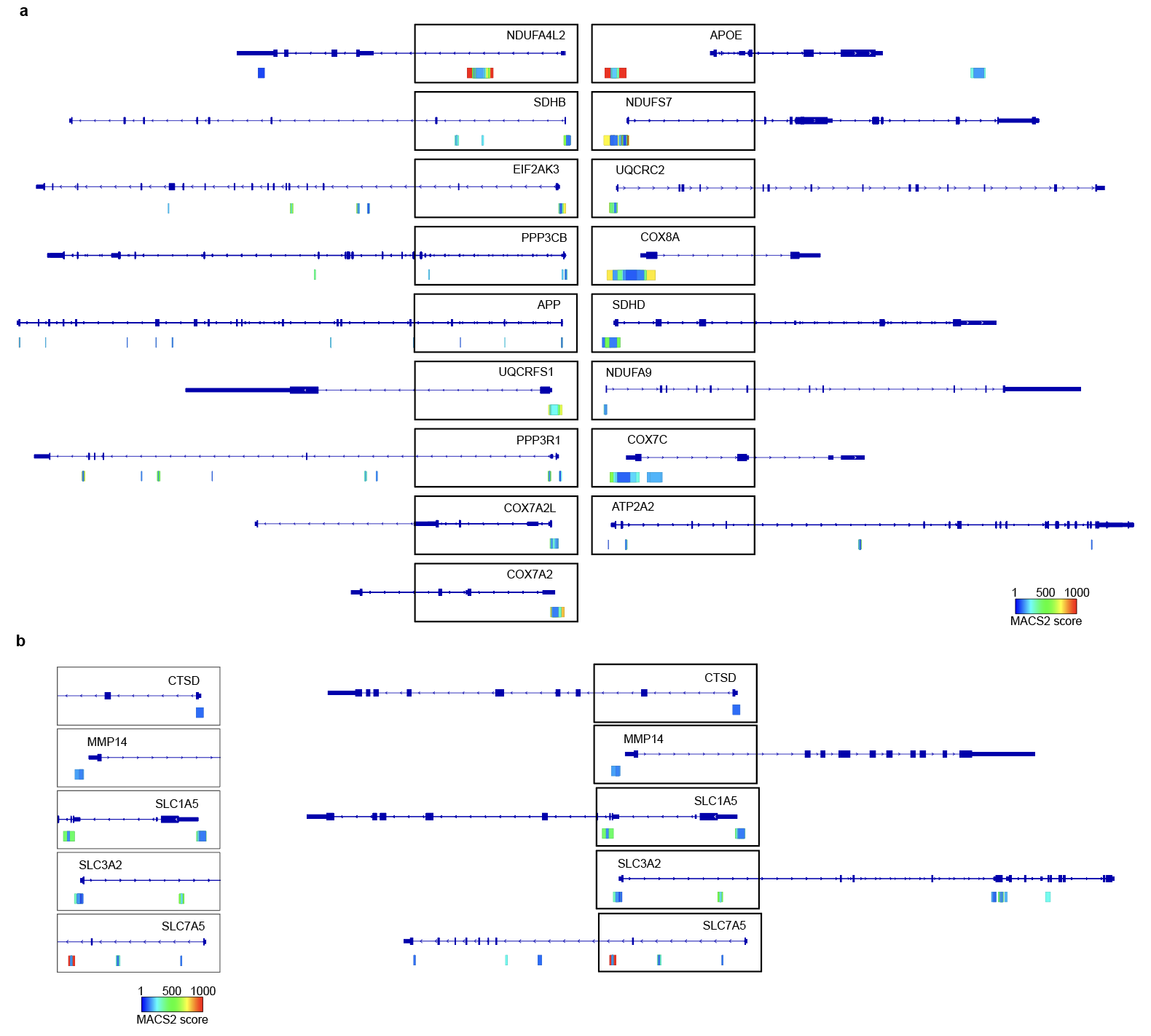


**Supplementary Fig. 9. Uncropped ChIP-Atlas analysis.**

Serum response factor (SRF) binds to AD-related genes (**a**) and amino acid metabolism-related genes, *CTSD, MMP14, SLC1A5, SLC3A2*, and *SLC7A5*. (**b**) Gene-protein binding intensity determined by model-based analysis of ChiP-Seq version 2 (MACS2) scores > 50. *CTSD,* Cathepsin D; *MMP,* matrix metalloproteinase*; SLC,* Solute carrier.

**
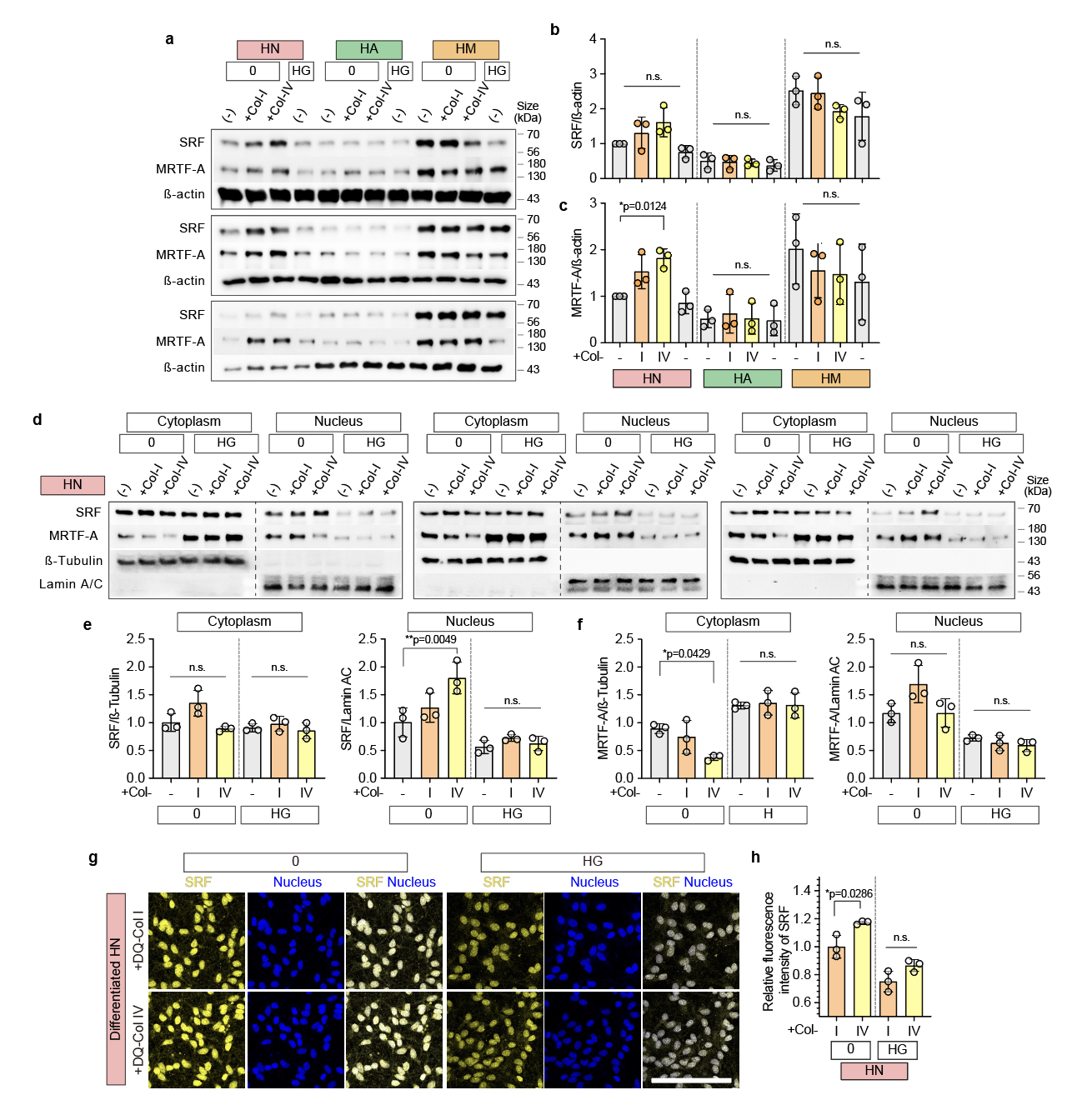
**

**Supplementary Fig. 10. Effects of ECM on SRF and MRTF activation in 2D hypoglycemic models.**

**a,** Expression of total SRF and MRTF-A proteins induced by DQ-collagen types I and IV under starvation (0) and fed (HG) conditions in HN, HA, and HM cultured on 2D plates. ß-actin was used as a loading control. Western blots of proteins in triplicate samples. **b, c,** Comparison of total SRF **(b)** and MRTF-A **(c)** protein expression in HNs incubated in 2D models without (-) DQ-collagen under starved (0) conditions. Graphs show the relative expression of SRF and MRTF-A normalized by ß-actin. Data in scatter dot plots shows means ± SD with bars and error bars. Significance was calculated using an ordinary one-way analysis of variance, followed by Tukey’s multiple comparison test (n=3; n.s.; not significant). **d,** Expression of total SRF and MRTF-A proteins by treating DQ-collagen types I and IV without (0) and with glucose (HG) in HNs in a 2D model. Loading controls for cytoplasm and nucleus were ß-Tubulin and Lamin A/C, respectively. Western blot analysis was performed in triplicate. **e, f,** Quantification of SRF (**e**) and MRTF-A **(f**) protein expression in cytoplasm and nucleus compared to non-treated (-) DQ-collagen under starved (0) conditions in HN-cultured 2D models. Graphs show relative expression of cytoplasmic and nuclear SRF and MRTF-A normalized by ß-Tubulin and Lamin A/C, respectively. Data in scatter dot plots are shown as means ± SD with bars and error bars. Significance was calculated using an ordinary one-way analysis of variance, followed by Tukey’s multiple comparison test (n=3; n.s.; not significant). **g,** Immunofluorescence staining of SRF (yellow) under DQ-collagen type I- and IV-treated starvation (0) and fed (HG) conditions in the HN-cultured 2D models. Nuclei are stained with DAPI (blue). Scale bar = 100 µm. **h,** Image-based quantification of the relative fluorescence intensity of SRF under DQ-collagen type I- and IV-treated starvation (0) and fed (HG) conditions in the HN-cultured 2D models. Graphs represented the relative values compared to the DQ-collagen I-treated starved conditions. Data in scatter dot plots show ± SD with bars and error bars. Significance was calculated using an ordinary one-way analysis of variance, followed by Tukey’s multiple comparison test (n=3; n.s.; not significant).


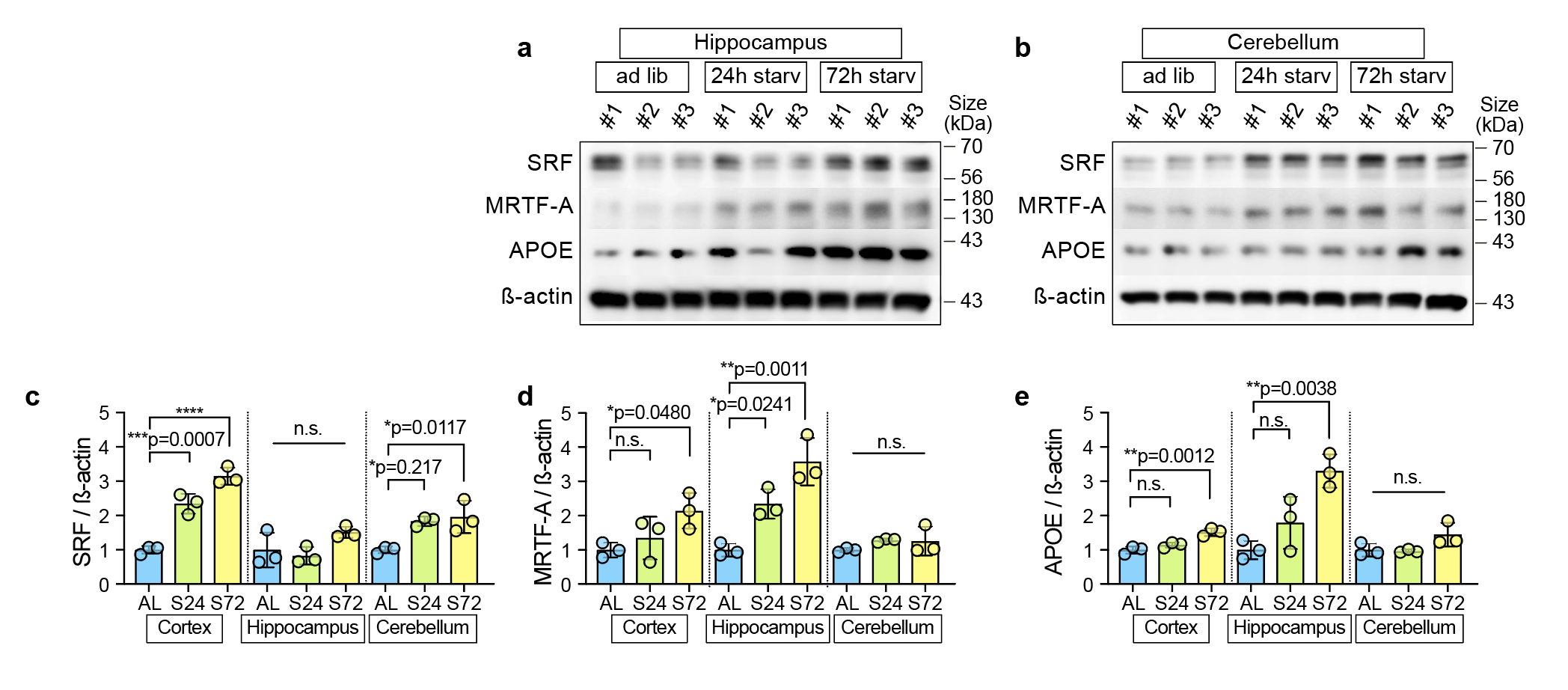


**Supplementary Fig. 11. Expression of SRF and MRTF in the starved mouse brains.**

**a-c,** Expression of SRF, MRTF-A, and APOE proteins under glucose *ad libitum* (AL), 24 hours (S24), and 72 hours of starvation (S72) conditions in the hippocampus (**a**), and cerebellum (**b**) regions in the brains of mice. ß-actin was used as a loading control. **c-e,** Relative protein expression compared to control AL conditions in cerebral cortex, hippocampus, and cerebellum. Graphs show expression of SRF **(c)**, MRTF **(d)**, and APOE **(e)** proteins under AL, S24, and S72 conditions in cerebral cortex, hippocampus, and cerebellum. Data on scatter dot plot are shown as means ± SD with bars and error bars (n=3; ****p < 0.0001, Ordinary one-way ANOVA, followed by Tukey multiple comparison tests. n.s.; not significant).


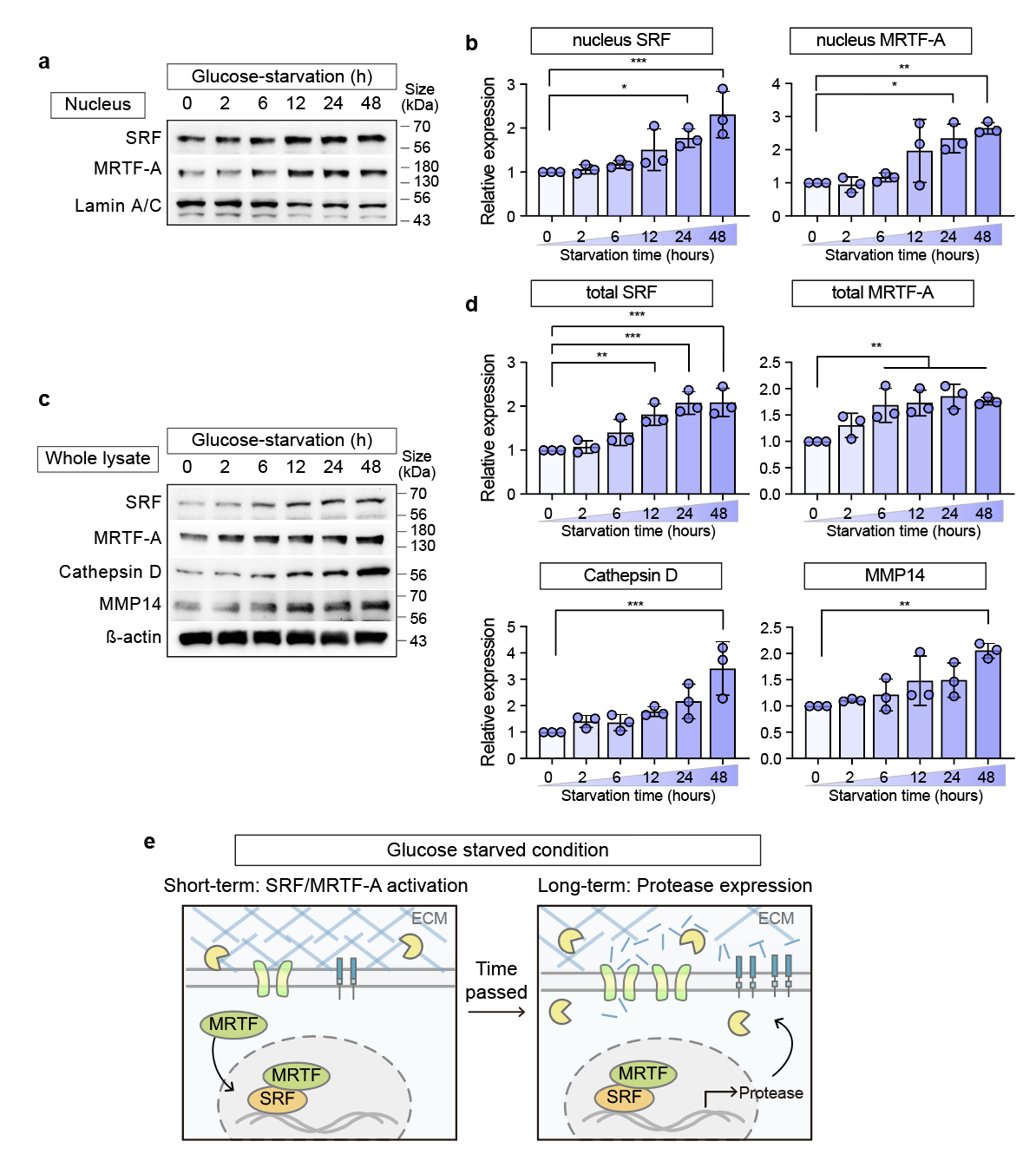


**Supplementary Fig. 12. Time-course analysis of protein expression changes in response to glucose starvation.**

**a, b,** Expression of nuclear SRF and MRTF-A during time-variable starvation for 2, 6, 12, 24, and 48 h. Lamin A/C was used as a loading control for the nuclear proteins. **(b**) Relative expression of nuclear SRF and MRTF-A (n =3). **c, d,** Levels of total SRF, MRTF-A, Cathepsin D, and MMP14 proteins under time-variable glucose depletion. ß-actin was used as a loading control. **(d**) Relative expression of total proteins (n = 3). **e**, Schematic illustration of the signaling mechanism during glucose depletion. In short-term starvation, SRF and MRTF-A are first activated in response to glucose-depleted conditions, then act as transcription factors for proteases involved in metabolic reprogramming.


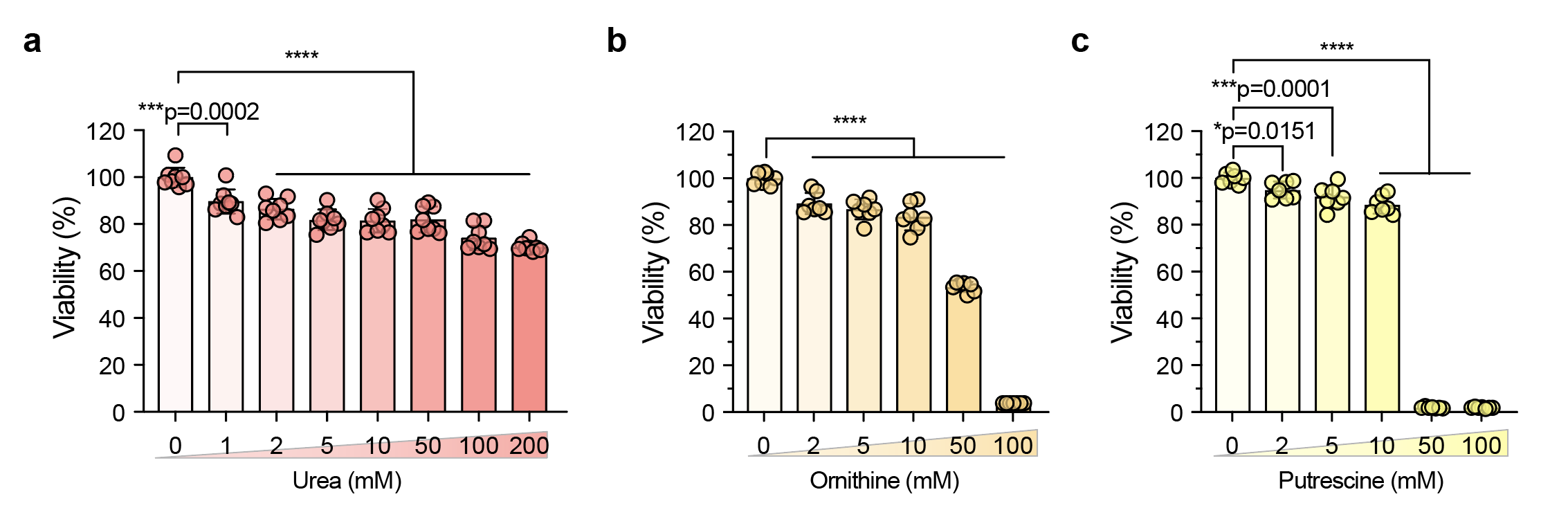


**Supplementary Fig. 13. Cell viability after incubation with urea, ornithine, and putrescine.**

Cells in HN-cultured 3D models were treated with urea (**a**) at concentrations from 0 to 200 mM, and with Orn (**b**) and Put (**c**) at concentrations from 0 to 100 mM.

**
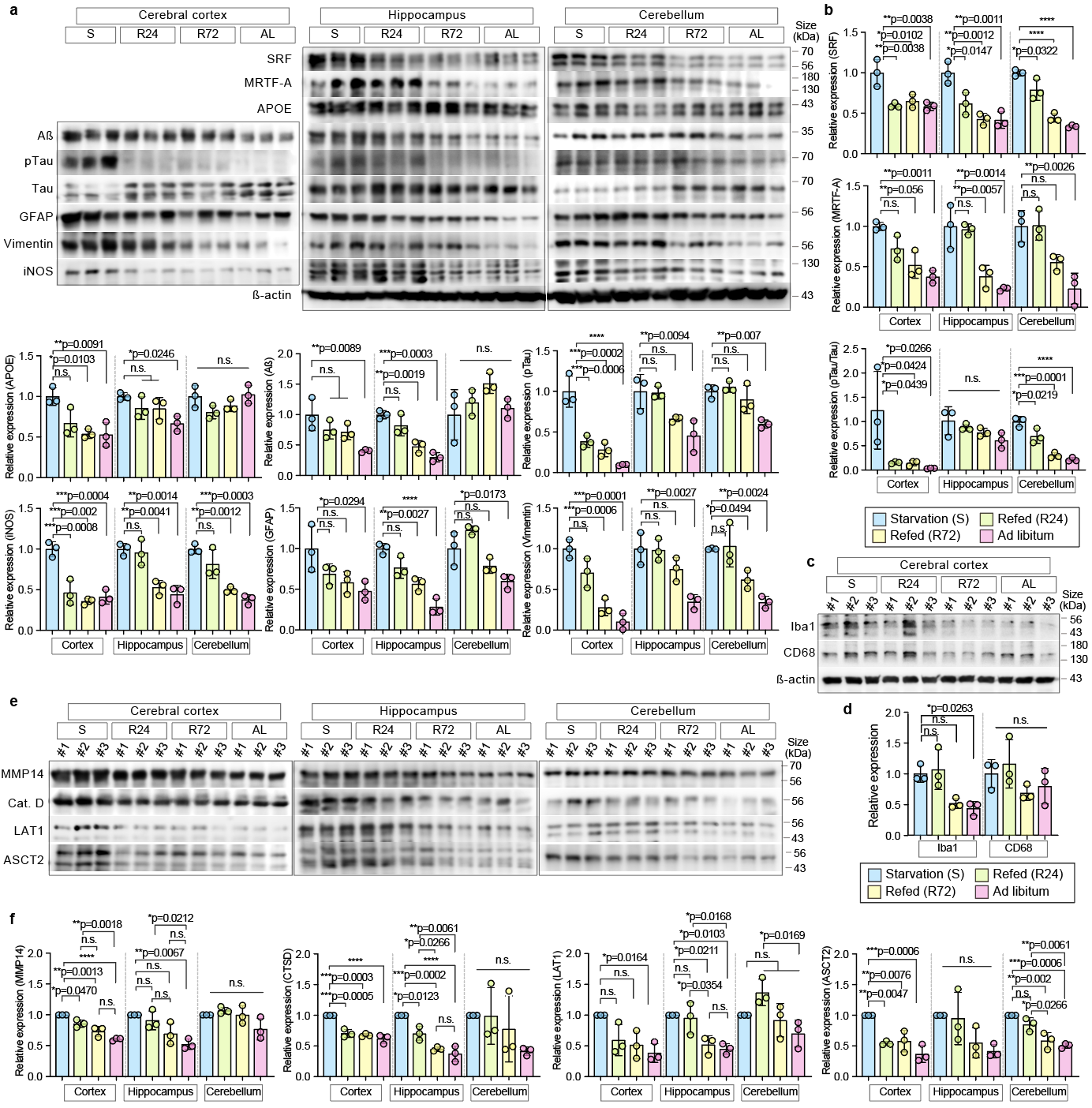
**

**Supplementary Fig. 14. Recovery of AD pathogenesis after refeeding via SRF/MRTF-A in the mouse brains.**

**a,** Expression of the SRF, MRTF-A, APOE, Aß, pTau, Tau, GFAP, Vimentin, and iNOS proteins in the cerebral cortex, hippocampus, and cerebellum regions of mice brains after time-variable refeeding for 24 (R24) to 72 hours (R72) compared to the starvation (S) and *ad libitum* (AL) conditions. ß-actin was used as a loading control. **b,** Quantitative protein expression values of SRF, MRTF-A, APOE, Aß, pTau, pTau/Tau, GFAP, Vimentin, and iNOS in the cerebral cortex, hippocampus, and cerebellum regions of mice brains after R24 and R72 compared to the S and AL conditions. **c,** Expression of the Iba1 and CD68 proteins in the cerebral cortex region of mice brains after R24 and R72 compared to the S and AL conditions. ß-actin was used as a loading control. **d,** Quantitative protein expression values of Iba1 and CD68 in the cerebral cortex regions of mice brains after R24 and R72 compared to the S and AL conditions. **e,** Expression of the MMP14, Cathepsin D, LAT1, and ASCT2 proteins in the cerebral cortex region of mice brains after R24 and R72 compared to the S and AL conditions. ß-actin was used as a loading control, which is identical to that in **a**. **f,** Quantitative protein expression values of MMP14, cathepsin D, LAT1, and ASCT2 in the cerebral cortex, hippocampus, and cerebellum regions of mice brains after R24 and R72 compared to the S and AL conditions. Graphs in **b, d,** and **f** show relative expression by comparing with S. Data in scatter dot plots in **b, d,** and **f** show means ± SD with bars and error bars. Ordinary one-way ANOVA followed by Tukey multiple comparison tests found no significant differences (n = 3; n.s., not significant).
